# Supplementary material for: The Dm-Myb Oncoprotein Contributes to Insulator Function and Stabilizes Repressive H3K27me3 PcG Domains
Source: Cell Rep. Author manuscript; Available in PMC 2020 Apr 21. (PMC7172335; doi:10.1016/j.celrep.2020.02.053)
Supplement: 1 [file NIHMS1574785-supplement-1.pdf]

Cell Reports, Volume *30*

Supplemental Information

The Dm-Myb Oncoprotein Contributes to Insulator Function and Stabilizes Repressive H3K27me3 PcG Domains

Juan F. Santana, Mrutyunjaya Parida, Abby Long, Joshua Wankum, Anthony J. Lilienthal, Krishna M. Nukala, and J. Robert Manak

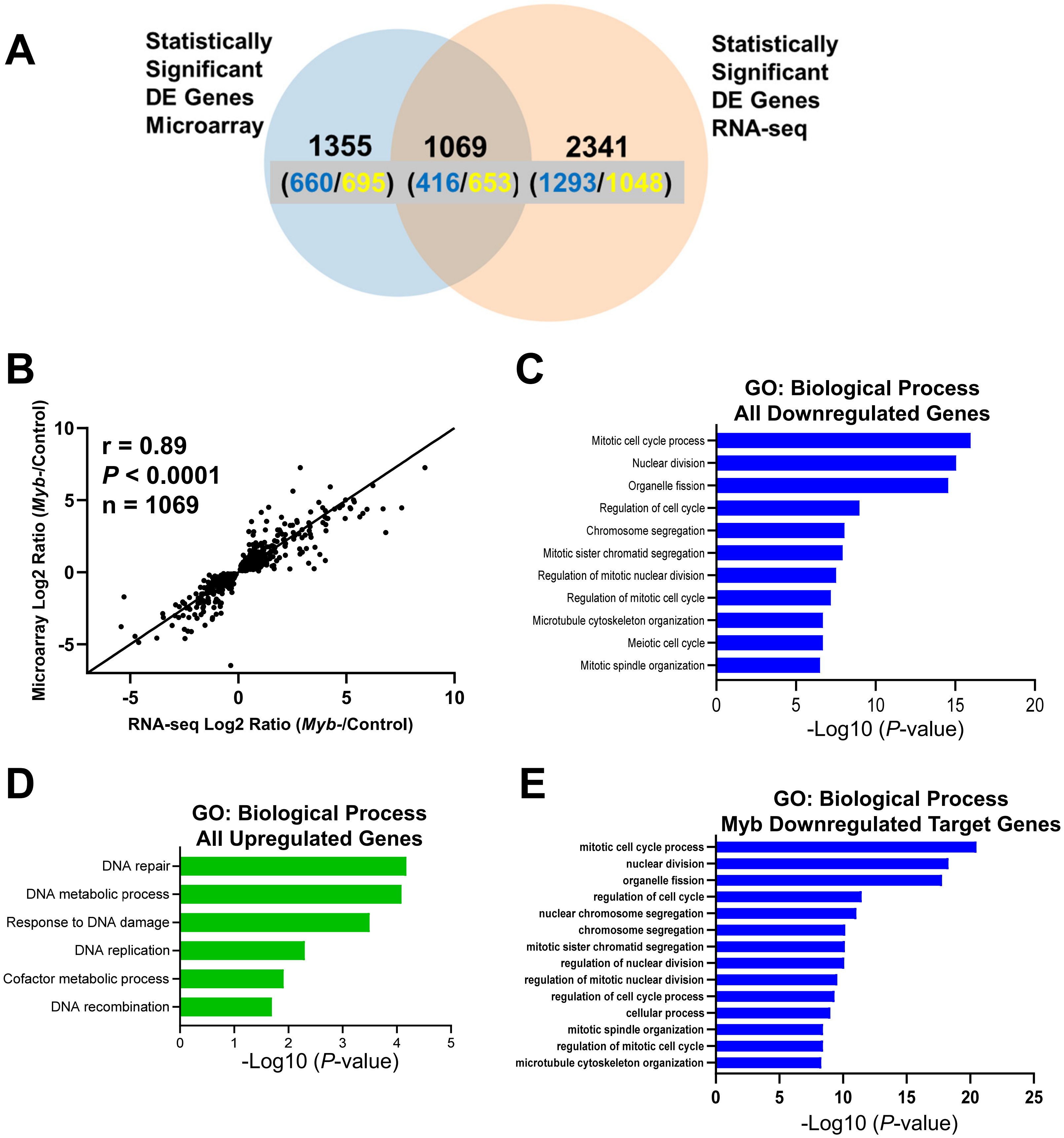

Figure S1. Comparison of gene expression microarray with RNA-seq data and gene ontology analysis of differentially expressed genes in the *Myb* mutant. Related to Figure 1. (A) Venn diagram comparison of statistically significant differentially expressed genes (control vs. *Myb-*) called by array ($P < 0.001$) and RNA-seq ($P < 0.05$). Numbers in blue denote downregulated genes in *Myb* mutant relative to control; numbers in yellow denote upregulated genes in *Myb* mutant relative to control. DE = differentially expressed. (B) Pearson correlation of the overlap of statistically significant differentially expressed genes called by both array and RNA-seq. (C) List of enriched biological processes associated with downregulated genes in the *Myb* mutant relative to control. (D) List of enriched biological processes associated with upregulated genes in the *Myb* mutant relative to control. (E) List of enriched biological processes associated with downregulated genes that are direct Myb targets in *Myb* mutant relative to control.

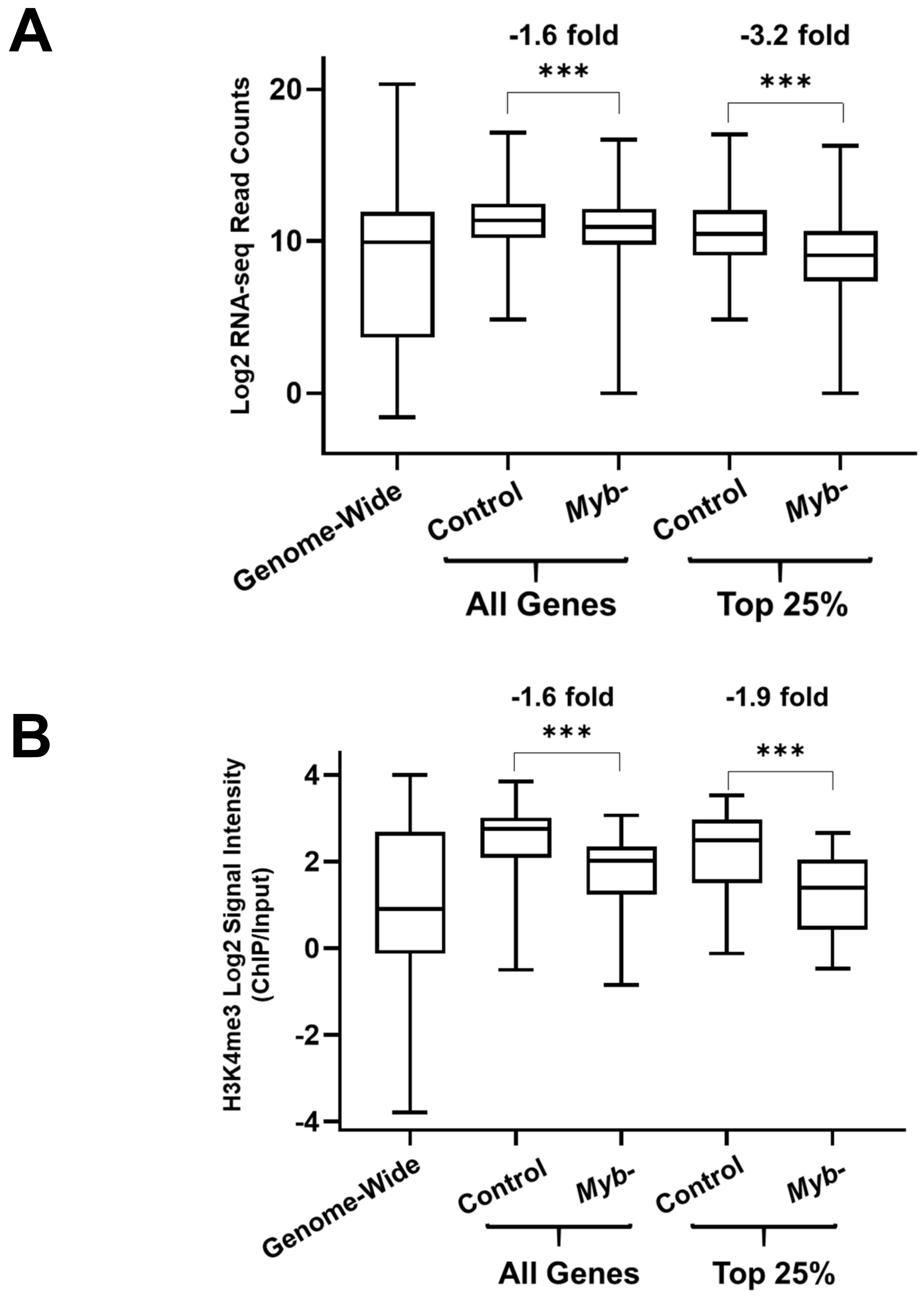

Figure S2. Direct Myb targets are highly expressed and potentiated by Myb. Related to Figure 1. (A) Comparison of expression levels of direct Myb-potentiated target genes in control and *Myb* mutants compared to gene expression levels genome-wide using RNA-seq data. (B) Comparison of H3K4me3 signal from TSS to 500 bp downstream for all direct Myb target genes in control and *Myb* mutants compared to genes genome-wide. Note that genes potentiated by Myb in (A) have higher median expression levels compared to genome-wide gene expression and that the absence of *Myb* leads to a significant reduction of gene expression (-1.6 median fold change). Also shown is the upper quartile (top 25%; highest fold change difference between *Myb* mutant and control) with a -3.2 median fold change. Reduced expression levels also correlate with reduced H3K4me3 (B; -1.6 median fold change between control and *Myb* mutant; -1.9 median fold change between control and *Myb* mutant for upper quartile genes). Boxes represent interquartile range (25th to 75th percentiles); whiskers represent min to max values; lines within boxes represent medians. All downregulated genes called as statistically significant by both array and RNA-seq. Genome-wide expression levels calculated using all annotated genes. List of genes used for (A) same as genes used for (B). ***$P < 0.0001$ for all comparisons, Mann-Whitney U test.

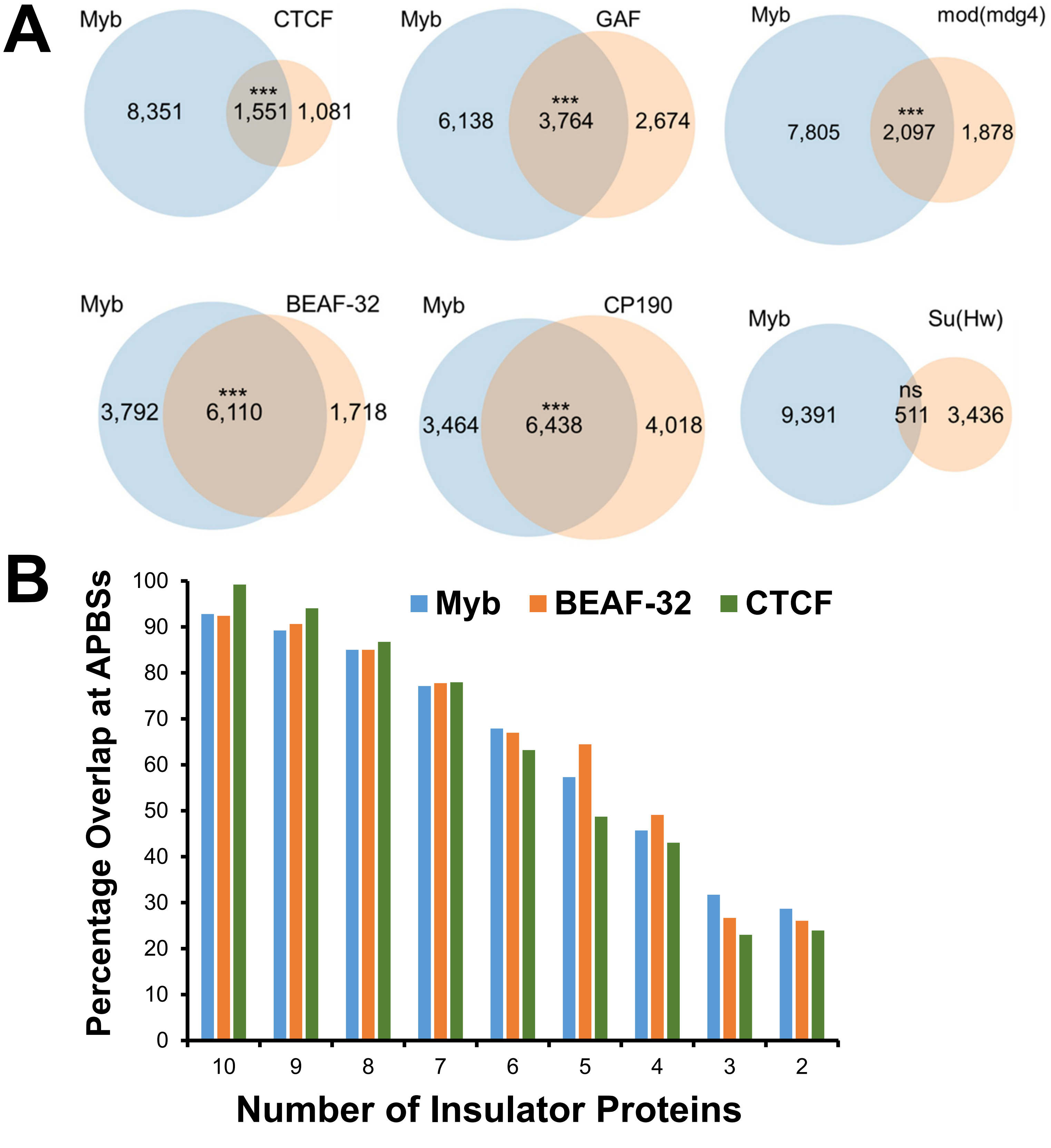

Figure S3. Overlap of Myb binding peaks with insulator protein peaks and high-occurrence architectural protein binding sites (APBS) genome-wide. **Related to Figure 2.** (A) Myb peaks show a statistically significant overlap with BEAF-32 (3.3-fold enrichment, $P < 2.15 \times 10^{-309}$), CP190 (2.8-fold enrichment, $P < 2.15 \times 10^{-309}$), dCTCF (2.3-fold enrichment, $P = 1.73 \times 10^{-132}$), GAF (2.6-fold enrichment, $P < 2.15 \times 10^{-309}$), and mod(mdg4) (2.4-fold enrichment, $P = 1.61 \times 10^{-174}$) peaks but show a negative enrichment for Su(Hw) peaks (0.48-fold enrichment, $P = 1.67 \times 10^{-55}$) and thus non-significant overlap. (B) Co-localization of Myb, BEAF-32, or dCTCF with increasing numbers of insulator proteins dCTCF, BEAF-32, Su(Hw), CP190, mod(mdg4), DREF, Chromator, l(3)mbt, TF3C, CAPH2. Note that colocalization is higher at sites when a maximal number of insulator proteins is present (10) and decreases at sites where fewer insulator proteins cluster. Binding site data for insulators obtained from modENCODE (see Results for sources). All comparisons, random permutation test. *** = statistical significance. ns = non-significance.

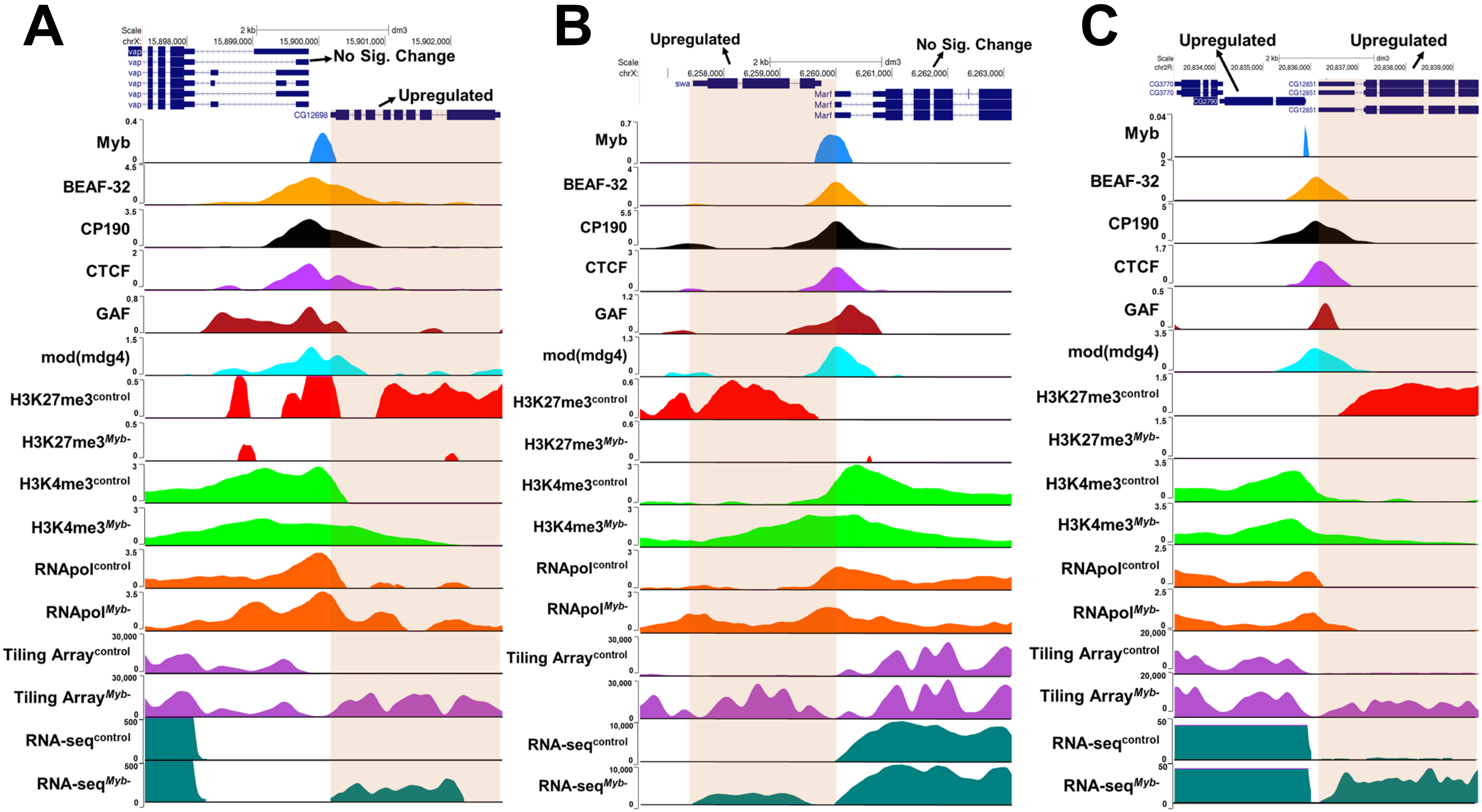

Figure S4. Additional examples of DPG-associated genes upregulated in Myb mutants. Related to Figure 2. (A) Loss of Myb binding between genes results in reduction of H3K27me3 and spreading of H3K4me3, with upregulation of *CG12698* (153 fold array, $P = 8.66 \times 10^{-32}$; 7.2-fold RNA-seq, $P = 1.08 \times 10^{-35}$). (B) Loss of Myb binding between genes results in reduction of H3K27me3 and spreading of H3K4me3, with upregulation of *swa* (64-fold array, $P = 1.26 \times 10^{-27}$; 74-fold RNA-seq, $P < 0.0001$). (C) Loss of Myb binding between genes results in reduction of H3K27me3 and spreading of H3K4me3, with upregulation of both *CG2790* (1.1-fold array, $P = 4 \times 10^{-5}$; 1.1-fold RNA-seq, $P < 0.05$) and *CG12851* (13-fold array, $P = 4.9 \times 10^{-19}$; 18-fold RNA-seq, $P = 1.6 \times 10^{-147}$).

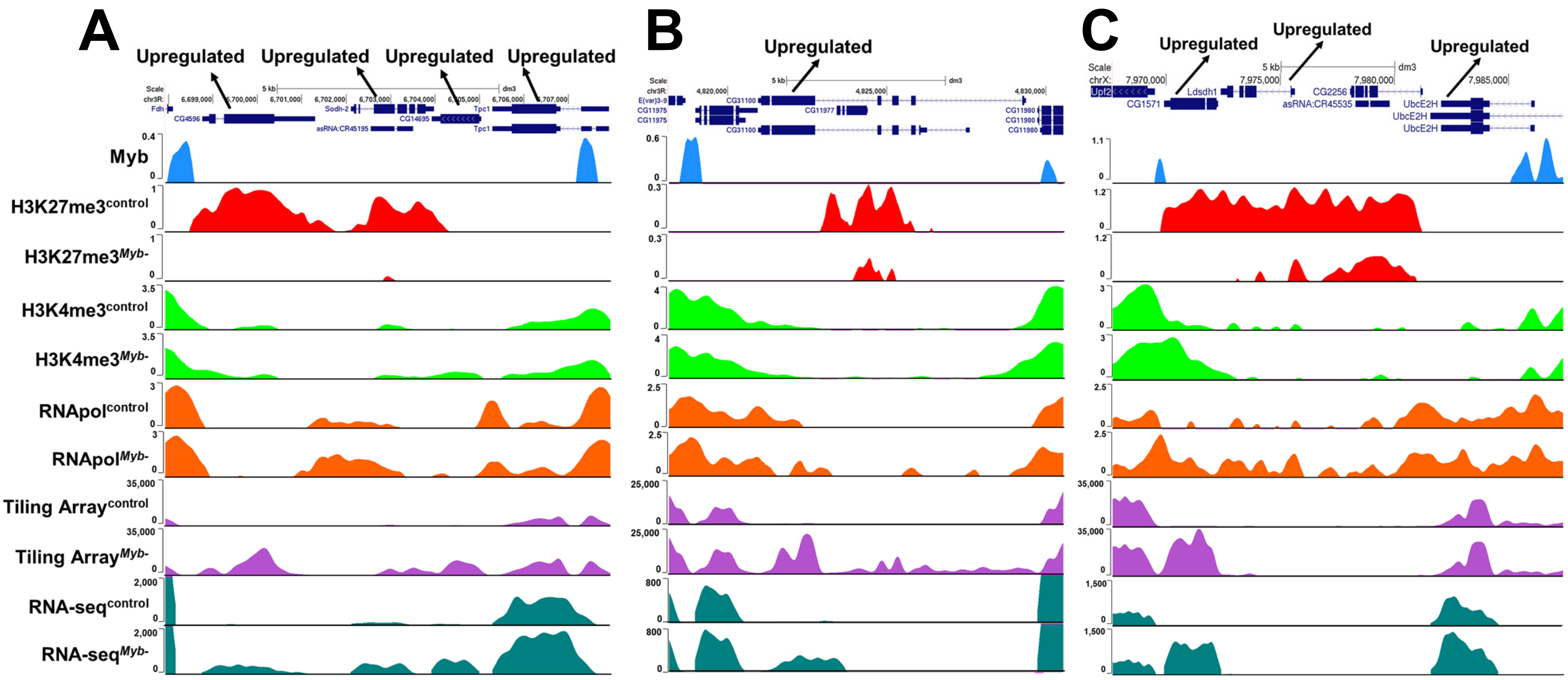

Figure S5. Additional examples of H3K27me3 domains demarcated by Myb. Related to Figure 3. (A) Loss of Myb binding results in reduction of H3K27me3, with upregulation of *CG4596* (22-fold array, P = 5.6 x 10^{-20}; 36-fold RNA-seq, P = 8.36 x 10^{-166}), *Sodh-2* (4.9-fold array, P = 8.69 x 10^{-14}; 4.3-fold RNA-seq, P = 1.93 x 10^{-41}), *CG14695* (14-fold array, P = 1.69 x 10^{-22}; 16-fold RNA-seq, P = 5.27 x 10^{-135}) and *Tpc1* (1.4-fold array, P = 1.85 x 10^{-11}; 1.5-fold RNA-seq, P = 7.78 x 10^{-22}) (B) Loss of Myb binding results in reduction of H3K27me3 and spreading of H3K4me3, with upregulation of *CG31100* (14-fold array, P = 5.68 x 10^{-26}; 4-fold RNA-seq, P = 4.09 x 10^{-16}). (C). Loss of Myb binding results in reduction of H3K27me3 and spreading of H3K4me3, with upregulation of *CG1571* (153-fold array, P = 5.77 x 10^{-26}; 397-fold RNA-seq, $P < 0.0001$), *CG2254* (2.4-fold array, P = 8.01 x 10^{-11}; 3-fold RNA-seq, P = 6 x 10^{-10}), and *UbcE2H* (1.1-fold array, P = 2.4 x 10^{-3}; 1.4-fold RNA-seq, P = 8.33 x 10^{-7}).
