# Supplementary material for: The Dm-Myb Oncoprotein Contributes to Insulator Function and Stabilizes Repressive H3K27me3 PcG Domains
Source: Cell Rep. Author manuscript; Available in PMC 2020 Apr 21. (PMC7172335; doi:10.1016/j.celrep.2020.02.053)
Supplement: 7 [file NIHMS1574785-supplement-7.pdf]

Cell Reports

Report

The Dm-Myb Oncoprotein Contributes to Insulator Function and Stabilizes Repressive H3K27me3 PcG Domains

Graphical Abstract

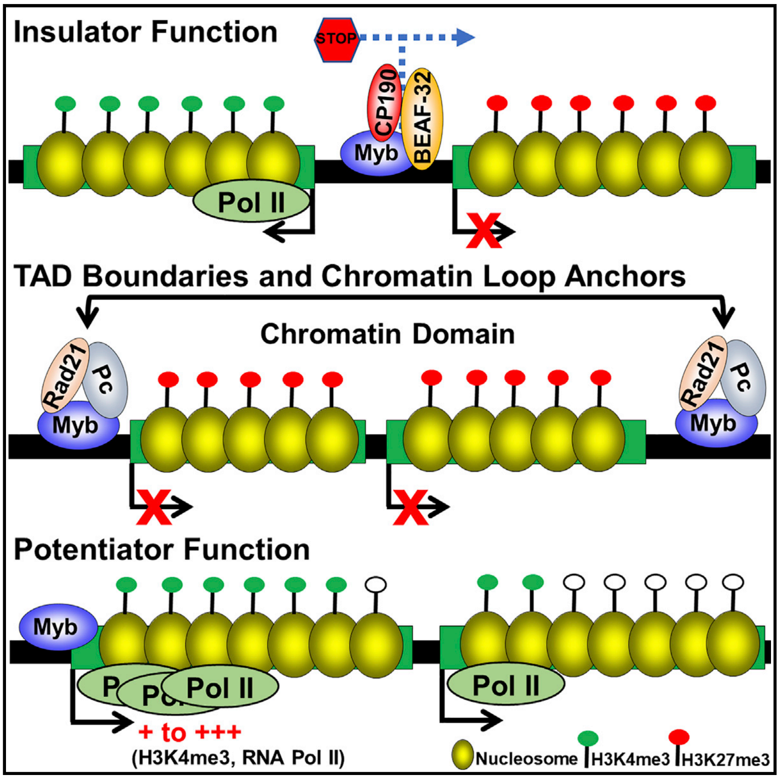

Highlights

- Myb transcriptional potentiation optimizes H3K4 methylation and RNA Pol II function
- Myb contributes to insulator function and promotes binding of BEAF-32 and CP190
- Myb is required to stabilize H3K27me3 domains genome wide
- Myb is enriched at TAD boundaries and chromatin loop anchors

Authors

Juan F. Santana, Mrutyunjaya Parida, Abby Long, Joshua Wankum, Anthony J. Lilienthal, Krishna M. Nukala, J. Robert Manak

Correspondence

john-manak@uiowa.edu

In Brief

Myb has been considered a transcriptional activator of primarily M phase genes. Here, Santana et al. show that Myb also contributes to insulator function, in part by promoting binding of insulator factors, and is required to stabilize repressive domains in the genome.

Santana et al., 2020, Cell Reports *30*, 3218–3228
March 10, 2020 © 2020 The Author(s).
https://doi.org/10.1016/j.celrep.2020.02.053

CellPress

Cell Reports
Report

The Dm-Myb Oncoprotein Contributes to Insulator Function and Stabilizes Repressive H3K27me3 PcG Domains

Juan F. Santana,[1,3] Mrutyunjaya Parida,[2,3] Abby Long,[3] Joshua Wankum,[3] Anthony J. Lilienthal,[3] Krishna M. Nukala,[3] and J. Robert Manak[1,2,3,4,5,*]

[1]Interdisciplinary Graduate Program in Genetics, University of Iowa, Iowa City, IA 52242, USA
[2]Interdisciplinary Graduate Program in Informatics, University of Iowa, Iowa City, IA 52242, USA
[3]Department of Biology, University of Iowa, Iowa City, IA 52242, USA
[4]Department of Pediatrics, University of Iowa, Iowa City, IA 52242, USA
[5]Lead Contact
*Correspondence: john-manak@uiowa.edu
https://doi.org/10.1016/j.celrep.2020.02.053

SUMMARY

Drosophila Myb (Dm-*Myb*) encodes a protein that plays a key role in regulation of mitotic phase genes. Here, we further refine its role in the context of a developing tissue as a potentiator of gene expression required for proper RNA polymerase II (RNA Pol II) function and efficient H3K4 methylation at promoters. In contrast to its role in gene activation, Myb is also required for repression of many genes, although no specific mechanism for this role has been proposed. We now reveal a critical role for Myb in contributing to insulator function, in part by promoting binding of insulator proteins BEAF-32 and CP190 and stabilizing H3K27me3 Polycomb-group (PcG) domains. In the absence of Myb, H3K27me3 is markedly reduced throughout the genome, leading to H3K4me3 spreading and gene derepression. Finally, Myb is enriched at boundaries that demarcate chromatin environments, including chromatin loop anchors. These results reveal functions of Myb that extend beyond transcriptional regulation.

INTRODUCTION

Vertebrates contain three representatives of the *Myb* gene family, consisting of A-, B-, and c-Myb, all of which encode transcription factors important for the proper expression of large numbers of genes (Rushton et al., 2003). *Drosophila* contains a single *Myb* gene (Dm-*Myb*) that is most similar to B-*Myb*. Dm-*Myb* mutants die before reaching adulthood at the 3rd instar/pupal stage, whereas mutation of *B-Myb* leads to early embryonic lethality in mice (Tanaka et al., 1999). Myb is part of the dREAM/Myb-MuvB (MMB) complex in flies, a large, conserved conglomerate of proteins that includes Rbf1/Rbf2, E2f2, Mips (40, 120, 130), DP, Lin-52, RPD3, l(3)mbt, and nucleosome remodeling factor (NURF) remodeling complex (Lewis et al., 2004). *Drosophila* dREAM plays a pivotal role in regulating proper expression of genes associated with the G2/M transition, and its absence leads to chromosomal instability and an increase in the mitotic index (Fung et al., 2002; Georlette et al., 2007; Manak et al., 2002; Wen et al., 2008). dREAM acts largely as a repressor through E2f2, whereas Myb predominantly functions as the activating arm of the complex (Georlette et al., 2007; Jackson et al., 2001; Lewis et al., 2004). All components of the complex are present at the majority of target promoters (Georlette et al., 2007), and the absence of both Myb and E2f2 causes variegated expression of a variety of genes (Wen et al., 2008). Several studies have implicated Myb as an epigenetic regulator of gene transcription (Bohla et al., 2014; Korenjak et al., 2014; Sim et al., 2012; Wen et al., 2008); however, no clear evidence exists regarding specific mechanisms by which this epigenetic regulation is achieved.

Insulator binding proteins play a major role in facilitating the proper regulation of gene expression (Misteli, 2007; Phillips-Cremins and Corces, 2013). Several exist in *Drosophila*, including the DNA binding factors *Drosophila* CTCF (dCTCF), suppressor of hairy wing [Su(Hw)], boundary-element-associated factor of 32kD (BEAF-32), and GAGA factor (GAF), and the ancillary proteins include centrosomal protein 190 (CP190) and modifier of mdg4 [mod(mdg4)]. Originally characterized by their ability to bind to regulatory sequences that can block promoter-enhancer crosstalk, insulator proteins are now known to play major roles not only as chromatin demarcating and enhancer-blocking barriers but also as key factors involved in the 3D arrangement of the genome within the nucleus into functional compartments known as topologically associating domains (TADs) (Phillips-Cremins and Corces, 2013). Active and repressed TADs are characterized by the enrichment of H3K4me3 and H3K27me3, respectively (Sexton et al., 2012). TADs are relatively stable across different cell types, but the sub-domains within are dynamic and parallel cell-type-specific gene expression (Dixon et al., 2012; Eagen et al., 2015; Dekker and Mirny, 2013).

In this study, we reveal several new roles for Myb in modulating gene expression and chromatin structure. First, we show that Myb is required for efficient H3K4 methylation and proper RNA polymerase II (RNA Pol II) dynamics at Myb target genes to potentiate gene expression. Second, we show that the loss of *Myb* leads to a genome-wide reduction of H3K27me3 in

 © 2020 The Author(s).
This is an open access article under the CC BY license (http://creativecommons.org/licenses/by/4.0/).

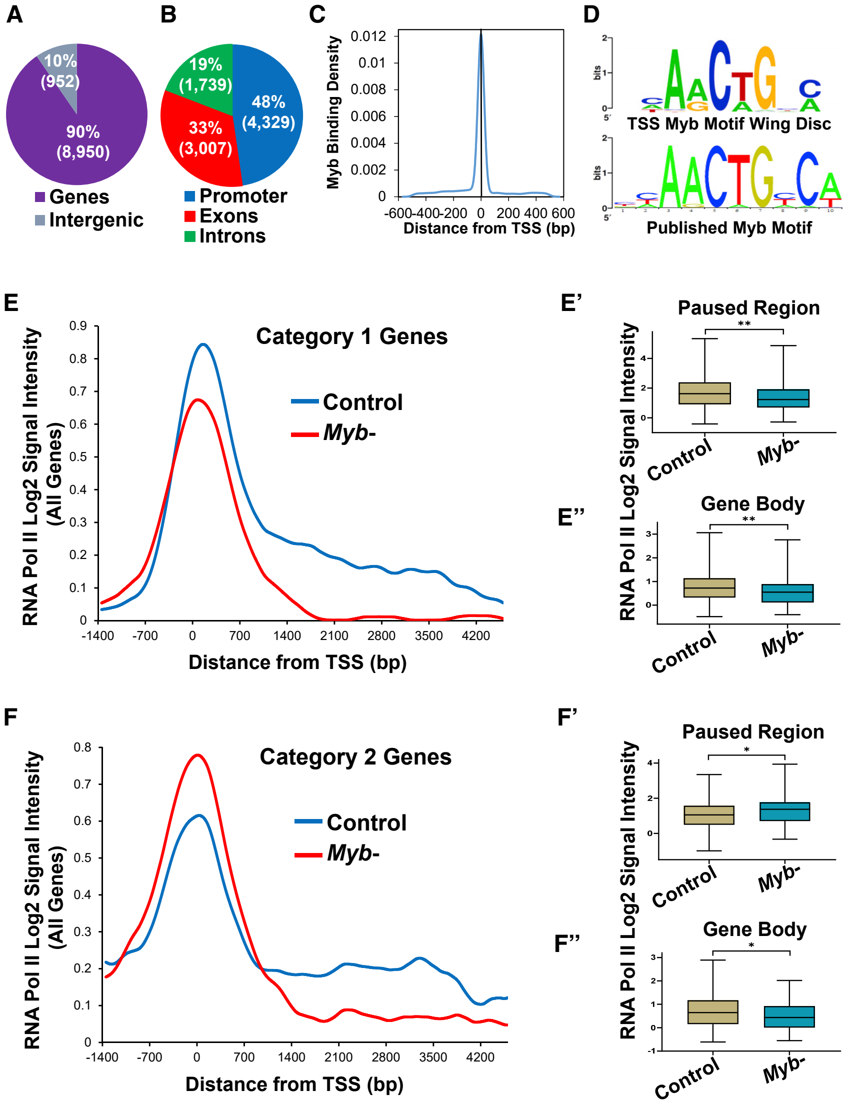

Figure 1. Myb Primarily Binds to TSS Regions to Promote Appropriate RNA Pol II Distribution at Target Genes

(A) ChIP-chip analysis of Myb in wing imaginal discs reveals its preference for binding genic regions, with 90% of the Myb peaks within gene boundaries ($p = 6.02 \times 10^{-13}$ compared to shuffled control).

(B) Breakdown of Myb binding within genes shows enrichment for promoter (−200 bp to TSS; $p = 5.79 \times 10^{-156}$ compared to shuffled control).

(C) The frequency of Myb binding peaks is highest at TSSs ($p = 1.75 \times 10^{-171}$ compared to shuffled control).

(D) Enrichment of the Myb binding motif of peaks present at a TSS ($p < 0.01$).

(E–F′) Genes potentiated by Myb show two distinct RNA Pol II distribution profiles as determined by averaging the RNA Pol II signal for all genes per category. Category 1 genes (E, 159 total) show reductions in RNA Pol II levels in *Myb* mutants compared to control at both the Pol II paused region ($p < 0.01$; E′) as well as across the gene body ($p < 0.01$; E″), whereas category 2 genes (F, 122 total) show an increase in RNA Pol II levels at the Pol II paused region ($p < 0.05$; F′) in addition to a reduction in Pol II levels across the gene body ($p < 0.05$; F″) in *Myb* mutants compared to control. Boxes represent interquartile range (25th to 75th percentiles; brown boxes control, blue boxes *Myb* mutant); lines within boxes represent medians. *$p < 0.05$, **$p < 0.01$, Mann-Whitney test.

repressive PcG domains, resulting in spread of the activating mark H3K4me3 and derepression of previously silent genes. Finally, we show that Myb is enriched at insulator sites and chromatin loop anchors, is required for the binding of insulator proteins CP190 and BEAF-32 to these sites, and is necessary for insulator function.

RESULTS

To identify tissue-specific genome-wide binding sites for Myb in an *in vivo* context, we performed chromatin immunoprecipitation followed by microarray hybridization (ChIP-chip) analysis using chromatin isolated from yw^{67} mitotically active late third instar mesothoracic wing discs. We identified 9,902 Myb peaks, with the vast majority mapping to genic regions (90%; $p = 6.02 \times 10^{-13}$ compared to shuffled control) (Figure 1A; Table S1). Of the binding sites in genic regions, the biggest category (48%; $p = 5.79 \times 10^{-156}$ compared to shuffled control) mapped to promoter regions, with most located at or near transcription start sites (Figures 1B and C; $p = 1.75 \times 10^{-171}$ compared to shuffled control). Motif analysis of the promoter sequences bound by Myb revealed enrichment for the consensus Myb DNA binding motif YAACKG ($p < 0.01$; Figure 1D).

To identify genes regulated by Myb in wing discs, we performed both gene expression microarray as well as RNA sequencing (RNA-seq) comparisons of Myb^{MH107} null mutants with yw^{67} controls (Table S2), finding a strong correlation of the datasets ($r = 0.89$; $p < 0.0001$; Figure S1) for overlapping differentially expressed genes. Heretofore, we used genes called as significantly changing in both array ($p < 0.001$) and RNA-seq ($p < 0.05$) for downstream analyses (1,069 total). Of these genes, 653 were upregulated (442 direct Myb targets), whereas 416 were downregulated (308 direct Myb targets). The largest enriched gene class of the direct Myb-activated targets was "cell cycle" (Figure S1), especially those in the mitotic class (G2/M or M), similar to previously published results (Georlette et al., 2007). Direct targets were modestly expressed in the absence of *Myb*, albeit at lower levels than when Myb was present (Figures S2A; $p < 0.0001$). Furthermore, Myb-activated genes were more transcriptionally active in the absence of *Myb* than genome-wide gene expression profiles (Figure S2A). This analysis revealed that Myb potentiates the expression of targets from a modest level of expression to a more robust level of expression.

Given the established epigenetic role of Myb in regulating gene expression (Wen et al., 2008), we assessed the genome-wide chromatin signatures of activating (H3K4me3) and repressive (H3K27me3) marks in both yw^{67} controls and *Myb* mutant wing discs. Overall, we found that H3K4me3 levels were elevated around Myb-bound transcription start sites (TSSs) of Myb-potentiated genes with optimal H3K4me3 levels dependent on Myb (Figure S2B). Because cell cycle genes make use of paused polymerases at their promoters to ensure a rapid transcriptional response (Core et al., 2008), we assessed RNA Pol II occupancy of direct Myb targets by ChIP-chip by using an antibody that recognizes RNA Pol II in both yw^{67} controls and *Myb* mutants. We analyzed two regions of each direct *Myb* target gene called as going down in *Myb* mutants (304 total; 4 removed as they were too short for gene body analysis): the RNA Pol II paused region (−30 to 300 bp downstream of the TSS) (Ebmeier et al., 2017) and the gene body (1,000 bp downstream of the TSS to the end of the gene). We found not only that Myb targets do indeed make use of paused RNA Pol II but also that there were two distinct RNA Pol II binding profiles in *Myb* mutants when comparing paused region occupancy with gene body occupancy. The majority of targets (159 genes; category 1; Figure 1E) showed reduced paused region occupancy ($p < 0.01$; Figure 1E′) as well as reduction in Pol II distribution across the gene body ($p < 0.01$; Figure 1E″) compared to controls, suggesting a decrease in efficient RNA Pol II recruitment and failure to undergo productive elongation. The second category of targets (122 genes; category 2; Figure 1F) showed increased paused region occupancy ($p < 0.05$; Figure 1F′) along with reduced Pol II distribution across the gene body ($p < 0.05$; Figure 1F″) compared to controls, suggesting an increase in RNA Pol II pausing and/or a failure to properly undergo elongation in the *Myb* mutant (thus leading to a buildup of RNA Pol II at the paused region). Therefore, although Myb is playing a role in RNA Pol II recruitment at some genes (the majority category), it is playing a role in RNA Pol II pause release and/or elongation for both groups.

Because we noticed that 10% of Myb binding sites were present in intergenic regions (Figure 1A), places where insulator proteins are known to bind (Felsenfeld et al., 2004), we overlapped our Myb ChIP-chip data with genome-wide binding site data mapped by the modENCODE project (Contrino et al., 2012; modENCODE Consortium et al., 2010; Nègre et al., 2011) for a variety of insulator proteins. In particular, given the *in vivo* nature of our study, we decided to use binding site data generated from 0- to 12-h embryos (Su(Hw), mod(mdg4), GAF, BEAF-32, and CP190), with the exception being dCTCF binding profiles, which were generated from S2 cells. Notably, 59% dCTCF (2.3-fold enrichment, $p = 1.73 \times 10^{-132}$), 58% GAF (2.6-fold enrichment, $p < 2.15 \times 10^{-309}$), 53% mod(mdg4) (2.4-fold enrichment, $p = 1.61 \times 10^{-174}$), and 13% Su(Hw) (p value not significant) binding sites were also occupied by Myb, with the greatest concordance of binding being with CP190 (62%; 2.8-fold enrichment, $p < 2.15 \times 10^{-309}$) and BEAF-32 (78%; 3.3-fold enrichment, $p < 2.15 \times 10^{-309}$) (Figure S3A; all p values generated using shuffled control). These data demonstrate that Myb binding is strongly associated with insulator proteins with the exception of Su(Hw).

To better explore the relationship between Myb and insulator proteins, we focused on divergently paired genes (DPGs) because insulator proteins as well as some dREAM complex members have been shown to be present at these sites (Korenjak et al., 2014; Nègre et al., 2010; Yang et al., 2012). We found that Myb bound to 84% of all *Drosophila* DPGs ($p = 2.32 \times 10^{-275}$ compared to shuffled control), similar to the percentage of DPGs bound by CP190 and BEAF-32 (each approximately 86%), but much higher than the binding observed for other insulator-associated factors (Figure 2A). Seventeen percent of the DPGs (360/2,101) showed statistically significant changes in gene expression in the absence of Myb (Figure 2B), with 340 (304 direct Myb targets) showing changes in expression of 1 of the 2 genes and 20 (18 direct Myb targets) showing changes in expression of both genes (Figure 2B). Finally, we found a statistically significant association of Myb binding to differentially expressed DPGs relative to bound unchanged DPGs ($p = 0.001$, Fisher's exact test). A representative example is shown in Figure 2C whereby the leftmost gene (*Upf2*) is located in an area devoid of the repressive mark H3K27me3 but is highly enriched for H3K4me3 and, thus, transcriptionally active (also see Figure S4). The absence of Myb leads to depletion of the H3K27me3 mark overlaying *CG1571*, with spreading of the H3K4me3 mark into *CG1571*, leading to an increase in its transcription. Moreover, the expression of *Upf2* remains at elevated levels in the *Myb* mutants. These data are consistent with a specific role for Myb in contributing directly to barrier insulator function but not in activation of the normally expressed gene (*Upf2)* of a DPG. Accordant with our data, we found that insulator elements shown to exhibit enhancer-blocking activity (Nègre et al., 2011; Schwartz et al., 2012) are bound by Myb, and genes directly adjacent to these elements show expression changes in the absence of Myb (Table S4).

A major role of insulator proteins is to compartmentalize functional domains (e.g., TADs) in the genome, defined in *Drosophila* as active, null, HP1-associated, and repressive PcG domains. We, thus, decided to assess the presence of Myb at borders of these domains (Sexton et al., 2012) and found that Myb was present at one or both boundaries of 99% of null domains, 99% of active domains, and 90% of HP1-associated domains (Table S4), with Myb present at over 93% of TAD boundary sites termed high-occupancy architectural protein binding sites (APBSs; occupied by 10 insulator-associated architectural proteins) (Van Bortle et al., 2014). Interestingly, Myb was also present at 99% of PcG H3K27me3 domains (Figure 2D; $p = 1.16 \times 10^{-32}$ compared to shuffled control). Myb signal increased at the start of each H3K27 domain, decreasing once inside the domain (Figure 2E). Remarkably, in *Myb* mutants, H3K27me3 levels were decreased across all PcG domains genome wide compared to controls (Figures 2F and 2F′). Finally, none of the genes encoding insulator proteins or other key chromatin-modifying enzymes are downregulated by the loss of Myb (Table S4), consistent with a role for Myb in establishing and/or stabilizing H3K27me3 PcG domains by binding to their boundaries.

To further explore genes upregulated in *Myb* mutants, we divided them into three categories depending on the levels of upregulation (moderate = 2- to 5-fold; high, >5- to 10-fold; maximal, >10-fold) and determined whether H3K27me3 depletion was coupled with H3K4me3 enrichment. We found that 16% of

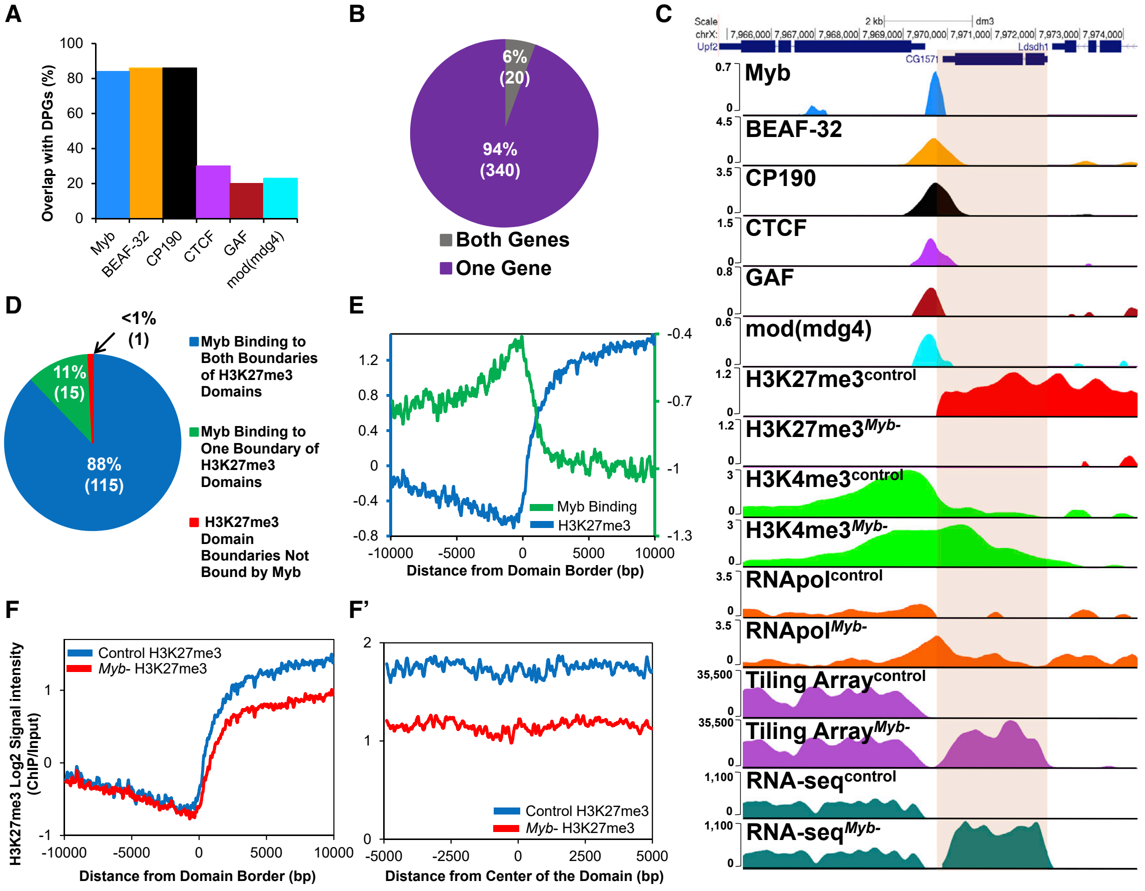

Figure 2. Myb Contributes to Insulator Function at DPGs and Binds to Boundaries of H3K27me3 PcG Domains to Maintain Domain Stability

(A) Binding of Myb and insulator proteins to promoter regions of DPGs. Myb binds to 84% of all DGPs present in the genome ($p = 2.32 \times 10^{-275}$ compared to shuffled control), comparable to BEAF-32 and CP190 (~86% for both).

(B) Absence of Myb leads to changes in expression of genes associated with 360 DPGs.

(C) Example of upregulation of expression in one gene of a DPG in *Myb* mutants normally bound by Myb and insulator proteins. Loss of Myb leads to the reduction of H3K27me3, leading to the spreading of H3K4me3 and higher RNA pol II occupancy, with upregulation of *CG1571*, as shown with tiling microarray data, gene expression microarray data (153-fold upregulation, $p = 5.77 \times 10^{-26}$), and RNA-seq data (397-fold upregulation, $p < 0.0001$).

(D) Myb is present at boundaries of 99% of all H3K27me3 domains previously described in *D. melanogaster* ($p = 1.16 \times 10^{-32}$ compared to shuffled control), with Myb binding to both boundaries of a domain 92% of the time.

(E–F′) Myb binding signal (purple line) is increased at boundaries of H3K27me3 domains (E; green line). Absence of Myb leads to reduced average levels of H3K27me3 at domain boundaries (F; red line) and continues across the length of the domains (F′; red line) ($p < 0.0001$, Student's t test). DE, differentially expressed. See STAR Methods for sources of binding data.

moderately upregulated genes in the *Myb* mutants (moderate category) showed depletion of H3K27me3 signal with no effect on H3K4me3 levels, whereas 1% of genes were depleted of H3K27me3 but enriched for H3K4me3 (Figure 3A). On the other hand, 27% of highly upregulated genes (high category) showed a reduction of H3K27me3 signal with no effect on H3K4me3 levels, whereas 3% of the genes had a reduction of H3K27me3 but enrichment for H3K4me3 (Figure 3A). The fraction of genes that showed both depletion of H3K27me3 as well as an increase of H3K4me3 increases 10-fold to 30% for maximally upregulated genes (maximal category, Figure 3A). ChIP-quantitative real-time PCR of randomly selected genes showing maximal upregulation in the *Myb* mutant (Figures 3B and 3C) all showed this reciprocal relationship. Representative examples are shown in Figures 3D and S5.

Given that Myb is required for the proper function of at least some insulators, we tested whether Myb might be playing a role in promoting the binding of insulator factors. We selected 22 insulator sites normally bound by Myb, BEAF-32, and CP190 as well as 3 insulator sites only bound by BEAF-32 and/or CP190 (but not Myb), as assessed by our Myb and BEAF-32/CP190 binding data (Contrino et al., 2012; modENCODE Consortium et al., 2010; Nègre et al., 2011), and we performed ChIP-quantitative real-time PCR of BEAF-32 and CP190 targets (the two insulator proteins with the strongest colocalizations with Myb) in both control and *Myb* mutant wing discs (Figure 4). Notably, for all sites tested that are normally bound by Myb, a decrease of BEAF-32 enrichment was observed in *Myb* mutants (Figure 4A) without reductions in BEAF-32 protein levels (Figure 4A′). A similar pattern was observed for CP190, with the

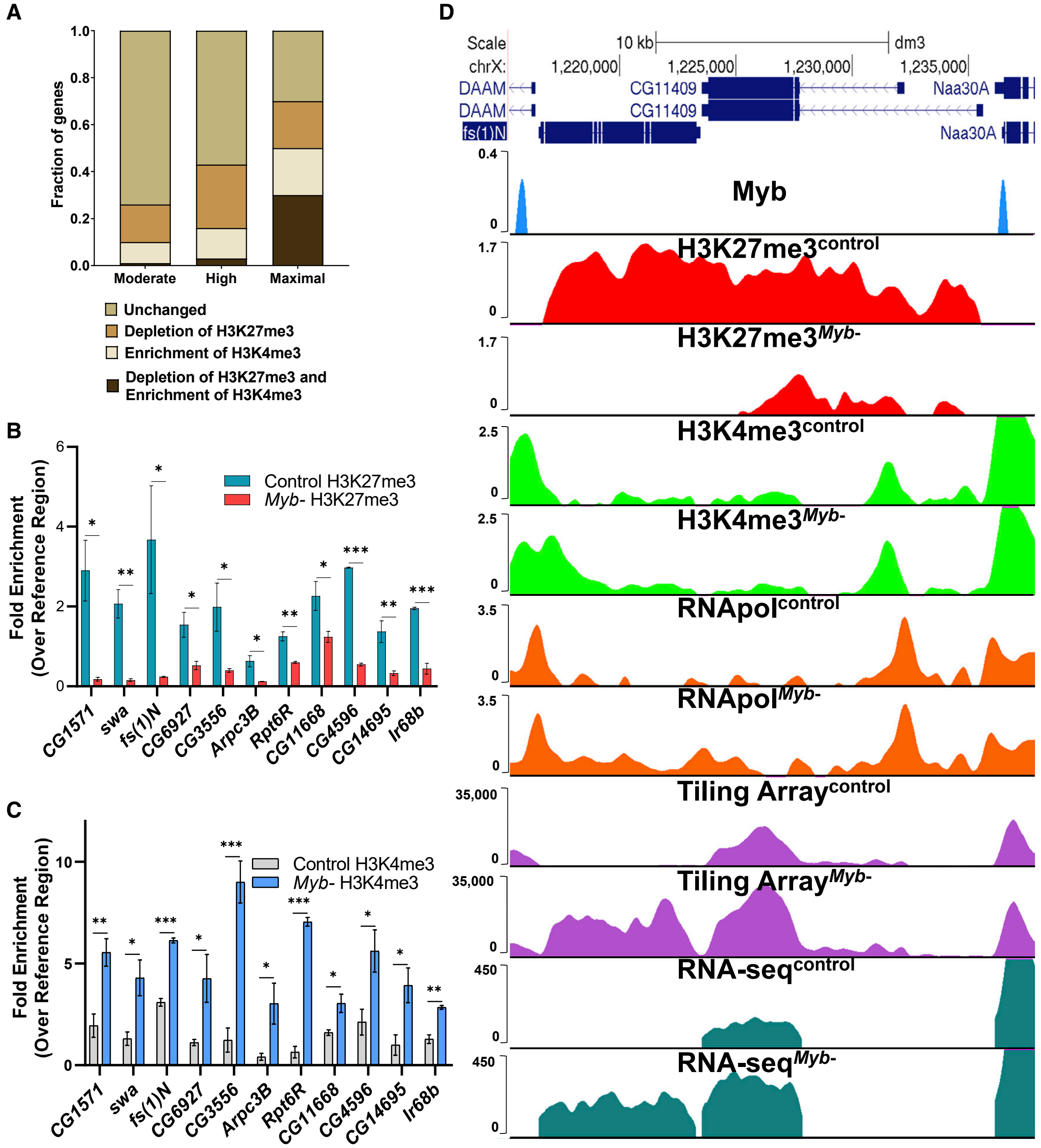

Figure 3. Loss of *Myb* Results in a Reduction of H3K27me3 along with Increased H3K4me3 and Upregulation of Genes within the Domain

(A) Genes upregulated in *Myb* mutants (moderate, high, and maximal) were subdivided depending on whether they showed a depletion of H3K27me3 only, an enrichment of H3K4me3 only, or a depletion of H3K27me3 and enrichment of H3K4me3 ($p < 0.05$, Student's t test). Genes not showing statistically significant changes were labeled as unchanged. Note that the most highly upregulated genes (maximal category) show the highest percentage of genes having both reduced H3K27me3 and increased H3K4me3 (30%) compared to the moderate or high categories (1% and 3%, respectively).

(B) ChIP-quantitative real-time PCR analysis of a selection of high to maximally upregulated genes reveals that H3K27me3 signal is reduced for all genes.

(C) ChIP-quantitative real-time PCR analysis of the same genes in (B) showing that H3K4me3 signal is increased for all genes.

(D) Representative example showing that loss of Myb leads to a reduction of H3K27me3 signal with H3K4me3 extension into *fs(1)N*, resulting in an increase of RNA Pol II occupancy and upregulation of transcription (array, ~22-fold upregulation, $p = 2.44 \times 10^{-23}$; RNA-seq, ~188 fold upregulation, $p < 0.0001$). Extension of H3K4me3 does not extend into *CG11409,* but the reduction of H3K27me3 leads to its overall upregulation (array, ~1.7-fold upregulation, $p = 1.01 \times 10^{-8}$; RNA-seq, ~2.2-fold upregulation, $p = 1 \times 10^{-32}$). For quantitative real-time PCR data, error bars represent mean ± SEM, *$p < 0.05$, **$p < 0.01$, two-tailed unpaired Student's t test.

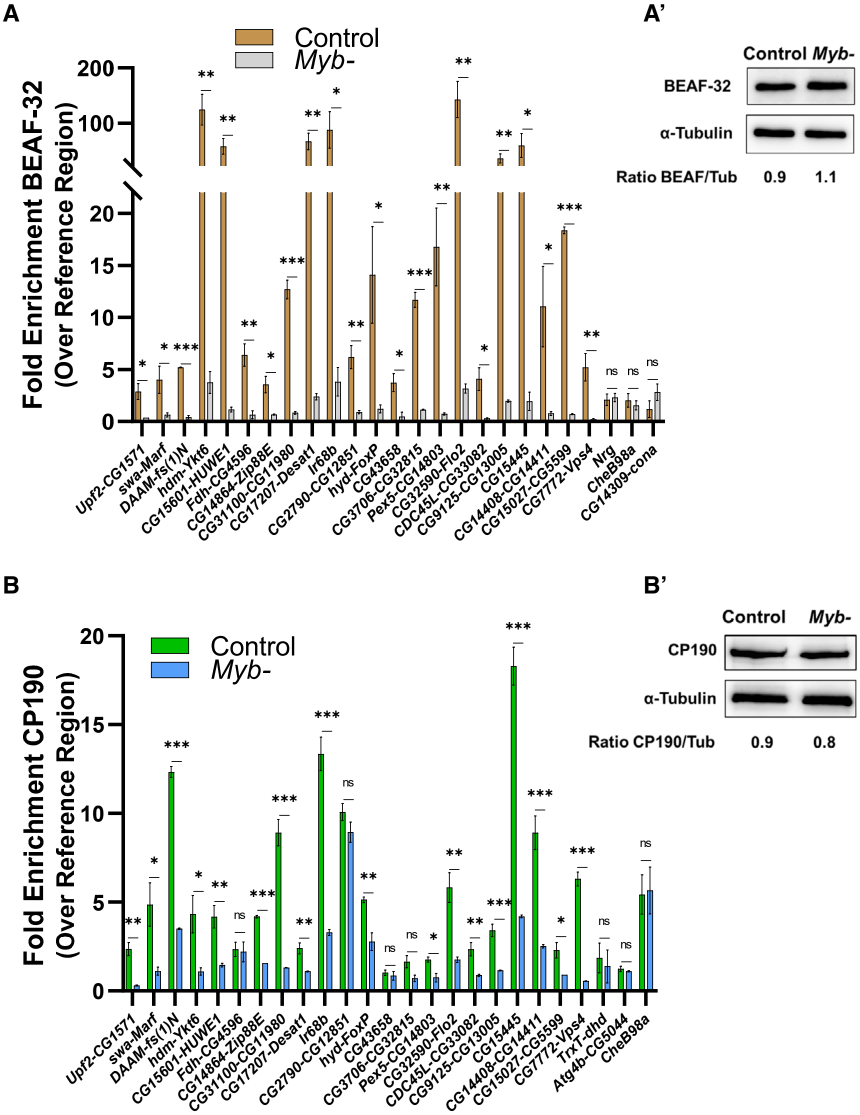

Figure 4. Myb Is Required for BEAF-32 and CP190 Binding at the Majority of Insulator Sites Tested

(A–B′) ChIP-quantitative real-time PCR analysis reveals that the loss of Myb leads to a significant reduction of BEAF-32 binding (A), even though the BEAF-32 protein expression level (A′) is comparable in the control and *Myb* mutant. Similarly, the loss of Myb leads to a significant reduction of CP190 binding for most insulator sites tested (B) even though CP190 protein expression level (B′) is comparable in the control and *Myb* mutant. Note that the last three sites depicted in (A) and (B) represent negative controls that overlap BEAF-32 and/or CP190 but not Myb peaks (*Nrg*, *CheB98a*, and *CG14309-cona* for BEAF-32; and *Trxt-dhd*, *Atg4b-CG5044*, and *CheB98a* for CP190). Gene pairs of DPGs are denoted by gene names separated by dashes. Error bars represent mean ± SEM; ns, not significant; *p < 0.05, **p < 0.01, ***p < 0.001, two-tailed unpaired Student's t test. ImageJ software was used to quantify western blot signals.

majority of sites (19, or 86%) showing decreases in binding (Figure 4B) in spite of comparable levels of CP190 protein in controls and *Myb* mutants (Figure 4B′). Finally, binding of BEAF-32/CP190 to insulators not targeted by Myb was not affected in *Myb* mutants (e.g., *Nrg* and *CheB98a* and DPGs *CG14309-cona*, *Trxt-dhd*, and *Atg4b-CG5044*). Next, we analyzed several insulator sites adjacent to genes upregulated in *Myb* mutants (Table S5A) and identified Myb binding sites at the majority of them (17 out of 19; 89%, p < 0.001), whereas none contained E2f2 binding sites. To confirm that E2f2 is not playing a role in the repression of these genes in wing discs, we randomly selected five genes and performed qRT-PCR from *E2f2* mutant wing discs. Similar to Kc167 cells, none of these genes were significantly upregulated in the *E2f2* mutant (Table S5B). These data suggest that targeting to insulator sites, as well as repression of adjacent genes, primarily requires Myb and not E2f2/Rbf. Collectively, these data indicate that Myb is required for the efficient binding of BEAF-32 and CP190 to Myb-targeted insulators and that Myb might contribute to insulator function, at least in part, by recruiting insulator factors. Further supporting this idea, Gurudatta and colleagues (Gurudatta et al., 2012) found 535 downregulated and 299 upregulated genes of DPGs in *BEAF-32* mutant wing discs. Interestingly, 25% (93) of the differentially expressed genes of DPGs in *Myb* mutants are also differentially expressed in *BEAF-32* mutants (with all changes in expression moving in the same direction for both mutants). A hypergeometric test confirmed that the overlap between differentially expressed genes of DPGs in the *Myb* and *BEAF* mutants is significant (p = 0.006).

Although we found that Myb binds to a high percentage of TAD boundaries (see Figure 5A for an example; high-resolution chromosome conformation capture [Hi-C] map image obtained from Chorogenome Navigator; Ramirez et al., 2018), recent work in flies has shown that chromatin loop anchors can also be contained within TADs (Cubeñas-Potts et al., 2017; Eagen et al., 2017; Ogiyama et al., 2018). These studies have identified several components of loop anchors, including the cohesin subunit Rad21 (Cubeñas-Potts et al., 2017; Eagen et al., 2017), Polycomb (Pc; Eagen et al., 2017; Ogiyama et al., 2018), and dCTCF (Cubeñas-Potts et al., 2017; Eagen et al., 2017), with Eagen et al. (2017) finding that Pc is enriched at loop anchors identified in Kc167 cells. Given that Myb is strongly associated with PcG repressive domains and is enriched at dCTCF sites, we thus decided to overlap our Myb peaks with the loop anchor points (Eagen et al., 2017). Notably, Myb peaks show a striking correlation with loop anchors, with an overall

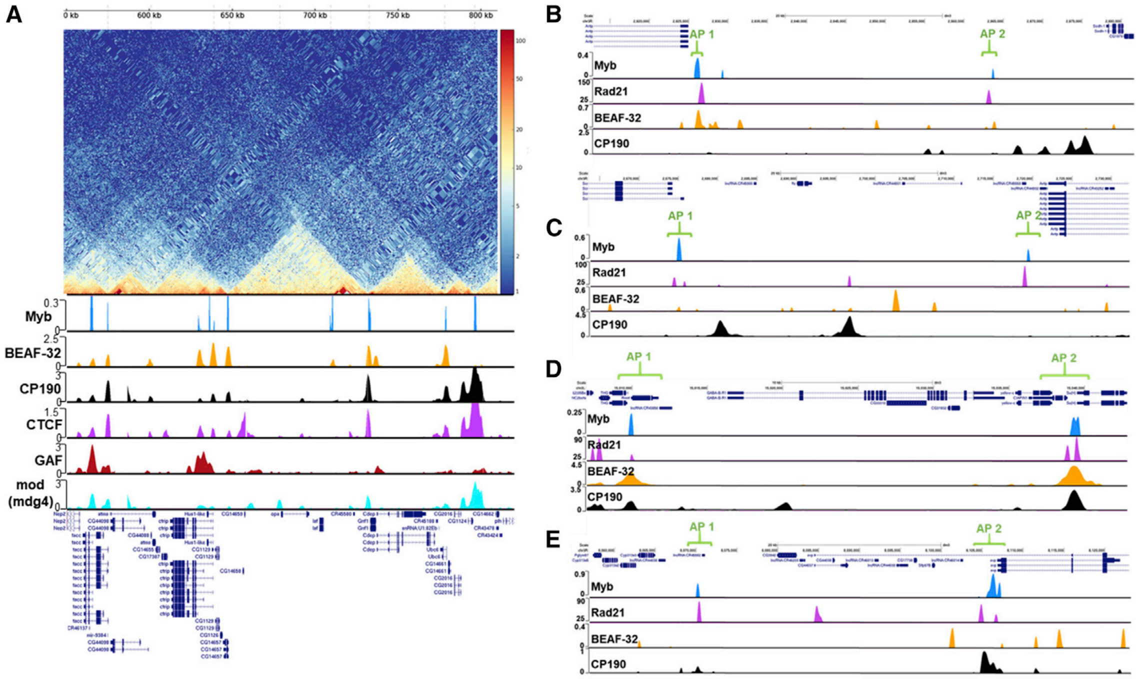

Figure 5. Myb Demarcates Boundaries of Topologically Associating Domains (TADs)

(A) Top: Hi-C map obtained from Chorogenome Navigator of a portion of chromosome 3R depicting the frequency of contacts between genomic loci (see text for details). Bottom: shown are binding sites for Myb, BEAF-32, CP190, dCTCF, GAF, and mod(mdg4). Note the colocalization of Myb with TAD boundaries as well as other insulator proteins.

(B–E) Myb binds to confirmed anchor points of chromatin loops upstream of *Antp* (B), between *Scr* and *Antp* (C), encompassing *GABA-B-R1* (D), and upstream of *svp* (E). Note the colocalization of Myb with the cohesin subunit Rad21 for all loop anchor regions. See STAR Methods for sources of binding data.

overlap of 65% ($p = 1.70 \times 10^{-17}$ compared to shuffled control) or 49% if only considering anchors with Myb peaks overlapping both contact points of an anchor (see Figures 5B–5E for examples; Rad21 track from Li et al., 2015; all other tracks from modENCODE Consortium et al., 2010). We then asked whether Myb had a statistically significant overlap with other known loop anchor components both genome-wide as well as at anchor points specifically using available binding site datasets (see STAR Methods). We find that Myb is enriched at Pc sites (23.2% genome wide, $p = 5.99 \times 10^{-12}$; 27.9% considering all anchors, $p = 7.08 \times 10^{-15}$), dCTCF sites (59% genome-wide, $p = 1.73 \times 10^{-132}$; 15.4% considering all anchors, $p = 3.01 \times 10^{-7}$) (Contrino et al., 2012; modENCODE Consortium et al., 2010; Nègre et al., 2011), and Rad21 binding sites (56.2% genome wide, $p < 2.15 \times 10^{-309}$; 40% considering all anchors, $p = 8.82 \times 10^{-26}$) (Van Bortle et al., 2014). Notably, if considering only anchors bound by dCTCF or Rad21, Myb binding is found at 73% and 75% of them, respectively. Collectively, these data demonstrate that Myb is enriched at, and significantly overlaps with, known components of chromatin anchor points.

DISCUSSION

In this study, we identify four novel roles for the *Drosophila* Myb oncoprotein. First, we show that Myb acts as a potentiator of target gene transcription, not merely as an epigenetic factor that maintains activated expression. Potentiation is associated with increased H3K4me3 as well as recruitment and pause release/elongation of RNA Pol II. Next, we show that in addition to binding promoter potentiator sites, Myb binds to insulator sites genome wide, is necessary to promote the binding of BEAF-32 and CP190 at Myb-shared sites, and is required for the function of at least a subset of insulators. Third, we show that Myb plays a critical role in formation and/or stabilization of H3K27me3 domains, preventing silent genes within these domains from becoming derepressed, in stark contrast to Myb's well-known role as an activator of gene expression. Finally, we show that Myb is enriched at TAD boundaries and chromatin loop anchors.

Similar to what has previously been reported, we identified an enrichment of cell cycle genes directly regulated by Myb, with the majority being G2/M but also several S phase genes (consistent with studies on B-Myb, supporting its role in S phase; Lam et al., 1992; Werwein et al., 2012). Notably, a recent study reported that Cyclin A (the cyclin that regulates aspects of both S phase and G2/M) directly binds to Myb and is required for expression of many of its targets (Rotelli et al., 2019). Further supporting a role for Myb in transcriptional regulation, we find that Myb is required for optimal levels of H3K4 promoter methylation, with Myb playing a key role in RNA Pol II dynamics. Analysis of the promoter regions of Myb-potentiated targets in *Myb*

mutants revealed two distinct classes of targets: one that requires Myb for efficient recruitment of RNA Pol II and both that use Myb for efficient RNA Pol II pause release/elongation. Myb may use NURF to establish a nucleosomal-free region that allows recruitment of RNA Pol II, similar to what has been observed with GAF at several targets (Duarte et al., 2016; Okada and Hirose, 1998; Tsukiyama et al., 1994; Tsukiyama and Wu, 1995). Further suggesting a critical role for *Myb* in transcriptional regulation, the failure to potentiate key targets in *Myb* mutants (e.g., *okr* and *Rad9* for S phase; *Cap-D2* for chromosome condensation; *mad2*, *Mps1*, *Pen*, *dgt6*, and *msd5* for M phase) likely explains the varied phenotypes observed in *Myb* mutants (including increased mitotic index, partially condensed chromosomes, aneuploidy, and S phase defects) (Fung et al., 2002; Manak et al., 2002).

Recent studies have shown that several components of dREAM can bind insulator sites (Bohla et al., 2014; Korenjak et al., 2014), and RNAi depletion of Mip40, Mip130, and E2f2 resulted in impairment of enhancer-blocking function at specific sites (Bohla et al., 2014). Furthermore, components of dREAM (Myb, Mip120, Mip130, Rbf1, E2f2, and DP) were shown to directly interact with dCTCF and/or CP190 (Bohla et al., 2014; Korenjak et al., 2014). Interestingly, double knockdown of dCTCF and CP190 resulted in loss of dREAM (Mip40, Mip120, Mip130, and E2f2) at some shared sites (Bohla et al., 2014). The results presented here further extend these studies and demonstrate that Myb directly contributes to boundary insulator function (including sites located at DPGs). Intriguingly, in many cases where one gene of a DPG is transcribed and the other is silent, we find that Myb has no role in expression of the activated gene; rather, Myb prevents inappropriate activation of the silent gene. This cannot be explained by loss of the primary repressive arm of the Myb complex (E2f2/Rbfs) because knockdown of E2f2 or Rbf1/2 in Kc167 cells led to derepression of only 17 out of the 96 genes upregulated when Myb is knocked down (Georlette et al., 2007). Furthermore, out of the 46 genes upregulated in SL2 cells upon knockdown of E2f2 or Rbf1/2 (Dimova et al., 2003), only 3 are upregulated in *Myb* mutant wing discs. Finally, the absence of E2f2 in 3rd instar larvae led to upregulation of genes from four DPGs (Korenjak et al., 2014); yet, these genes are not upregulated in *Myb* mutant wing discs. Of the insulator sites we have shown to require Myb, the vast majority (89%) contain Myb but not E2f2 consensus sites, similar to what was observed in Kc167 cells for genes requiring Myb for repression (Georlette et al., 2007).

Consistent with Myb playing a role in insulators that separate different chromatin neighborhoods, we find a striking correlation between Myb occupancy and active, null, PcG H3K27me3 (all 99%), and APBS TAD boundaries (>93%; Van Bortle et al., 2014). TAD boundaries have been associated with chromatin looping, and studies on CTCF and CP190 in vertebrates and invertebrates, respectively (Bushey et al., 2009; Matthews and Waxman, 2018; Nora et al., 2017; Pekowska et al., 2018; Rao et al., 2014), have proposed or confirmed a role for these insulator factors in tethering the chromatin loops. Further work has shown that chromatin loop anchors can also exist within TADs (Cubeñas-Potts et al., 2017; Eagen et al., 2017; Ogiyama et al., 2018; Smith et al., 2016). It is particularly noteworthy that Myb occupies the majority of chromatin loop anchor points identified by Eagen et al. (2017), in addition to significant overlaps with several other chromatin factors (including dCTCF, Rad21, and Pc) at anchor points. Further work is needed to determine whether Myb plays a role in tethering chromatin loops, although it is intriguing to speculate that the loss of the H3K27me3 mark in *Myb* or *dCTCF* (Van Bortle et al., 2012) mutants could result from disrupting H3K27me3 loops, which, in turn, might diminish that region's ability to associate with an H3K27me3 nuclear subcompartment (Rao et al., 2014). The observation that dCTCF has been shown to directly interact with Myb further suggests that these two chromatin factors can collaborate (Bohla et al., 2014), and a remarkable 73% of the anchors bound by dCTCF are also bound by Myb (similar to the Rad21-bound anchors, for which 75% are also occupied by Myb). We postulate that B-Myb (the most closely related vertebrate Myb family member to *Dm*-Myb) and CTCF might be playing similar collaborative roles in humans (Cubeñas-Potts et al., 2017; Eagen et al., 2017; Pekowska et al., 2018).

Although a role for Myb in transcriptional activation has been recognized for some time, its role in insulator function is particularly intriguing, as this activity appears to be separate from its transcriptional role. Indeed, the chromatin changes we observe at many DPGs and other insulator elements strongly support the direct role of Myb in promoting a barrier function between chromatin states (in part by promoting binding of BEAF-32/CP190). However, unlike previously described examples of barrier insulator loss, which results in heterochromatin spreading into euchromatin (Bartkuhn et al., 2009; Dorman et al., 2007; Yang and Corces, 2011), we observe the opposite scenario in *Myb* mutants, namely the spreading of H3K4me3, which may be enabled by the significant loss of the H3K27me3 mark. This is the very definition of barrier insulator function, namely, maintaining a discrete separation of chromatin environments. Whether Myb is playing a role in chromatin loop formation is unclear at this point, but its overlap with the majority of loop anchor points identified in at least one study in flies is intriguing. Further work will be needed to address Myb's role at the loop anchors and whether this role relates to stabilization of H3K27me3 domains and/or barrier insulator function. For example, directed mutagenesis of Myb-dependent insulator sites by using tools, such as CRISPR-Cas9, to specifically target Myb, dCTCF, or BEAF-32 sites can provide additional insight into the respective roles of these insulator factors. In addition, assay for transposase-accessible chromatin using sequencing (ATAC-seq) or chromosome conformation capture (3C)-type techniques performed in both controls and *Myb* mutants can be used to determine whether important chromatin/3D structure changes might underlie the altered transcriptional phenotypes.

STAR★METHODS

Detailed methods are provided in the online version of this paper and include the following:

- KEY RESOURCES TABLE
- LEAD CONTACT AND MATERIALS AVAILABILITY

- EXPERIMENTAL MODEL AND SUBJECT DETAILS
  - *Drosophila* strains
- METHOD DETAILS
  - Microarray gene expression analysis
  - RNA-seq analysis
  - Gene Ontology analysis
  - Chromatin immunoprecipitation
  - ChIP-chip analysis
  - Motif discovery
  - RNA polymerase analysis
  - Upregulated genes for H3K4 and H3K27 significance analysis
  - Overlaps of Myb peaks with genomic elements
  - Random permutation testing
  - Quantitative real-time PCR analyses
  - Western blot analysis
- QUANTIFICATION AND STATISTICAL ANALYSIS
- DATA AND CODE AVAILABILITY

SUPPLEMENTAL INFORMATION

Supplemental Information can be found online at https://doi.org/10.1016/j.celrep.2020.02.053.

ACKNOWLEDGMENTS

We thank Dr. Victor Corces, Dr. Karen Adelman, and Dr. Michael Botchan for providing antibodies used in this study and Dr. Corces for fruitful and engaging discussions. This work was supported in part by National Institutes of Health Predoctoral Training Grant T32GM008629 and funds to J.R.M. from the University of Iowa.

AUTHOR CONTRIBUTIONS

J.F.S. and J.R.M. designed the study. J.F.S. performed ChIP-chip, ChIP-quantitative real-time PCR, gene expression and tiling microarray experiments, RNA-seq, and western blot. A.L. performed hybridization and scanning of microarrays. J.W. helped J.F.S. with larval dissections. M.P. performed the bioinformatics analysis. A.J.L. and K.M.N. performed crosses, larval dissections, and qRT-PCR. J.F.S, M.P., and J.R.M. analyzed and interpreted the data. J.F.S. and J.R.M. prepared the figures. J.F.S and J.R.M. wrote the manuscript.

DECLARATION OF INTERESTS

The authors declare no competing interests.

Received: April 2, 2019
Revised: September 30, 2019
Accepted: February 12, 2020
Published: March 10, 2020

REFERENCES

Bailey, T.L., Boden, M., Buske, F.A., Frith, M., Grant, C.E., Clementi, L., Ren, J., Li, W.W., and Noble, W.S. (2009). MEME SUITE: tools for motif discovery and searching. Nucleic Acids Res. *37*, W202–W208.

Bartkuhn, M., Straub, T., Herold, M., Herrmann, M., Rathke, C., Saumweber, H., Gilfillan, G.D., Becker, P.B., and Renkawitz, R. (2009). Active promoters and insulators are marked by the centrosomal protein 190. EMBO J. *28*, 877–888.

Blanton, J., Gaszner, M., and Schedl, P. (2003). Protein:protein interactions and the pairing of boundary elements *in vivo*. Genes Dev. *17*, 664–675.

Bohla, D., Herold, M., Panzer, I., Buxa, M.K., Ali, T., Demmers, J., Krüger, M., Scharfe, M., Jarek, M., Bartkuhn, M., and Renkawitz, R. (2014). A functional insulator screen identifies NURF and dREAM components to be required for enhancer-blocking. PLoS One *9*, e107765.

Bolger, A.M., Lohse, M., and Usadel, B. (2014). Trimmomatic: a flexible trimmer for Illumina sequence data. Bioinformatics *30*, 2114–2120.

Bushey, A.M., Ramos, E., and Corces, V.G. (2009). Three subclasses of a Drosophila insulator show distinct and cell type-specific genomic distributions. Genes Dev. *23*, 1338–1350.

Contrino, S., Smith, R.N., Butano, D., Carr, A., Hu, F., Lyne, R., Rutherford, K., Kalderimis, A., Sullivan, J., Carbon, S., et al. (2012). modMine: flexible access to modENCODE data. Nucleic Acids Res. *40*, D1082–D1088.

Core, L.J., Waterfall, J.J., and Lis, J.T. (2008). Nascent RNA sequencing reveals widespread pausing and divergent initiation at human promoters. Science *322*, 1845–1848.

Crooks, G.E., Hon, G., Chandonia, J.M., and Brenner, S.E. (2004). WebLogo: a sequence logo generator. Genome Res. *14*, 1188–1190.

Cubeñas-Potts, C., Rowley, M.J., Lyu, X., Li, G., Lei, E.P., and Corces, V.G. (2017). Different enhancer classes in Drosophila bind distinct architectural proteins and mediate unique chromatin interactions and 3D architecture. Nucleic Acids Res. *45*, 1714–1730.

Dekker, J., and Mirny, L. (2013). Biological techniques: Chromosomes captured one by one. Nature *502*, 45–46.

Dimova, D.K., Stevaux, O., Frolov, M.V., and Dyson, N.J. (2003). Cell cycle-dependent and cell cycle-independent control of transcription by the Drosophila E2F/RB pathway. Genes Dev. *17*, 2308–2320.

Dixon, J.R., Selvaraj, S., Yue, F., Kim, A., Li, Y., Shen, Y., Hu, M., Liu, J.S., and Ren, B. (2012). Topological domains in mammalian genomes identified by analysis of chromatin interactions. Nature *485*, 376–380.

Dorman, E.R., Bushey, A.M., and Corces, V.G. (2007). The role of insulator elements in large-scale chromatin structure in interphase. Semin. Cell Dev. Biol. *18*, 682–690.

Duarte, F.M., Fuda, N.J., Mahat, D.B., Core, L.J., Guertin, M.J., and Lis, J.T. (2016). Transcription factors GAF and HSF act at distinct regulatory steps to modulate stress-induced gene activation. Genes Dev. *30*, 1731–1746.

Eagen, K.P., Hartl, T.A., and Kornberg, R.D. (2015). Stable Chromosome Condensation Revealed by Chromosome Conformation Capture. Cell *163*, 934–946.

Eagen, K.P., Aiden, E.L., and Kornberg, R.D. (2017). Polycomb-mediated chromatin loops revealed by a subkilobase-resolution chromatin interaction map. Proc. Natl. Acad. Sci. USA *114*, 8764–8769.

Ebmeier, C.C., Erickson, B., Allen, B.L., Allen, M.A., Kim, H., Fong, N., Jacobsen, J.R., Liang, K., Shilatifard, A., Dowell, R.D., et al. (2017). Human TFIIH Kinase CDK7 Regulates Transcription-Associated Chromatin Modifications. Cell Rep. *20*, 1173–1186.

Felsenfeld, G., Burgess-Beusse, B., Farrell, C., Gaszner, M., Ghirlando, R., Huang, S., Jin, C., Litt, M., Magdinier, F., Mutskov, V., et al. (2004). Chromatin boundaries and chromatin domains. Cold Spring Harb. Symp. Quant. Biol. *69*, 245–250.

Fung, S.M., Ramsay, G., and Katzen, A.L. (2002). Mutations in Drosophila myb lead to centrosome amplification and genomic instability. Development *129*, 347–359.

Georlette, D., Ahn, S., MacAlpine, D.M., Cheung, E., Lewis, P.W., Beall, E.L., Bell, S.P., Speed, T., Manak, J.R., and Botchan, M.R. (2007). Genomic profiling and expression studies reveal both positive and negative activities for the Drosophila Myb MuvB/dREAM complex in proliferating cells. Genes Dev. *21*, 2880–2896.

Grant, C.E., Bailey, T.L., and Noble, W.S. (2011). FIMO: scanning for occurrences of a given motif. Bioinformatics *27*, 1017–1018.

Gurudatta, B.V., Ramos, E., and Corces, V.G. (2012). The BEAF insulator regulates genes involved in cell polarity and neoplastic growth. Dev. Biol. *369*, 124–132.

Jackson, J., Ramsay, G., Sharkov, N.V., Lium, E., and Katzen, A.L. (2001). The role of transcriptional activation in the function of the Drosophila myb gene. Blood Cells Mol. Dis. *27*, 446–455.

Korenjak, M., Kwon, E., Morris, R.T., Anderssen, E., Amzallag, A., Ramaswamy, S., and Dyson, N.J. (2014). dREAM co-operates with insulator-binding proteins and regulates expression at divergently paired genes. Nucleic Acids Res. *42*, 8939–8953.

Lam, E.W., Robinson, C., and Watson, R.J. (1992). Characterization and cell cycle-regulated expression of mouse B-myb. Oncogene *7*, 1885–1890.

Langmead, B., and Salzberg, S.L. (2012). Fast gapped-read alignment with Bowtie 2. Nat. Methods *9*, 357–359.

Lewis, P.W., Beall, E.L., Fleischer, T.C., Georlette, D., Link, A.J., and Botchan, M.R. (2004). Identification of a Drosophila Myb-E2F2/RBF transcriptional repressor complex. Genes Dev. *18*, 2929–2940.

Li, L., Lyu, X., Hou, C., Takenaka, N., Nguyen, H.Q., Ong, C.T., Cubeñas-Potts, C., Hu, M., Lei, E.P., Bosco, G., et al. (2015). Widespread rearrangement of 3D chromatin organization underlies polycomb-mediated stress-induced silencing. Mol. Cell *58*, 216–231.

Love, M.I., Huber, W., and Anders, S. (2014). Moderated estimation of fold change and dispersion for RNA-seq data with DESeq2. Genome Biol. *15*, 550.

Manak, J.R., Mitiku, N., and Lipsick, J.S. (2002). Mutation of the Drosophila homologue of the Myb protooncogene causes genomic instability. Proc. Natl. Acad. Sci. USA *99*, 7438–7443.

Manak, J.R., Wen, H., Van, T., Andrejka, L., and Lipsick, J.S. (2007). Loss of Drosophila Myb interrupts the progression of chromosome condensation. Nat. Cell Biol. *9*, 581–587.

Mathelier, A., Fornes, O., Arenillas, D.J., Chen, C.Y., Denay, G., Lee, J., Shi, W., Shyr, C., Tan, G., Worsley-Hunt, R., et al. (2016). JASPAR 2016: a major expansion and update of the open-access database of transcription factor binding profiles. Nucleic Acids Res. *44*, D110–D115.

Matthews, B.J., and Waxman, D.J. (2018). Computational prediction of CTCF/cohesin-based intra-TAD loops that insulate chromatin contacts and gene expression in mouse liver. eLife *7*, e34077.

Mi, H., Muruganujan, A., Ebert, D., Huang, X., and Thomas, P.D. (2019). PANTHER version 14: more genomes, a new PANTHER GO-slim and improvements in enrichment analysis tools. Nucleic Acids Res. *47*, D419–D426.

Misteli, T. (2007). Beyond the sequence: cellular organization of genome function. Cell *128*, 787–800.

modENCODE Consortium; Roy, S., Ernst, J., Kharchenko, P.V., Kheradpour, P., Negre, N., Eaton, M.L., Landolin, J.M., Bristow, C.A., Ma, L., et al. (2010). Identification of functional elements and regulatory circuits by Drosophila modENCODE. Science *330*, 1787–1797.

Nègre, N., Brown, C.D., Shah, P.K., Kheradpour, P., Morrison, C.A., Henikoff, J.G., Feng, X., Ahmad, K., Russell, S., White, R.A., et al. (2010). A comprehensive map of insulator elements for the Drosophila genome. PLoS Genet. *6*, e1000814.

Nègre, N., Brown, C.D., Ma, L., Bristow, C.A., Miller, S.W., Wagner, U., Kheradpour, P., Eaton, M.L., Loriaux, P., Sealfon, R., et al. (2011). A cis-regulatory map of the Drosophila genome. Nature *471*, 527–531.

Nien, C.Y., Liang, H.L., Butcher, S., Sun, Y., Fu, S., Gocha, T., Kirov, N., Manak, J.R., and Rushlow, C. (2011). Temporal coordination of gene networks by Zelda in the early Drosophila embryo. PLoS Genet. *7*, e1002339.

Nora, E.P., Goloborodko, A., Valton, A.L., Gibcus, J.H., Uebersohn, A., Abdennur, N., Dekker, J., Mirny, L.A., and Bruneau, B.G. (2017). Targeted Degradation of CTCF Decouples Local Insulation of Chromosome Domains from Genomic Compartmentalization. Cell *169*, 930–944.e922.

Ogiyama, Y., Schuettengruber, B., Papadopoulos, G.L., Chang, J.M., and Cavalli, G. (2018). Polycomb-Dependent Chromatin Looping Contributes to Gene Silencing during Drosophila Development. Mol. Cell *71*, 73–88.e75.

Okada, M., and Hirose, S. (1998). Chromatin remodeling mediated by Drosophila GAGA factor and ISWI activates fushi tarazu gene transcription *in vitro*. Mol. Cell. Biol. *18*, 2455–2461.

Pekowska, A., Klaus, B., Xiang, W., Severino, J., Daigle, N., Klein, F.A., Oles, M., Casellas, R., Ellenberg, J., Steinmetz, L.M., et al. (2018). Gain of CTCF-Anchored Chromatin Loops Marks the Exit from Naive Pluripotency. Cell Syst *7*, 482–495.e410.

Phillips-Cremins, J.E., and Corces, V.G. (2013). Chromatin insulators: linking genome organization to cellular function. Mol. Cell *50*, 461–474.

Quinlan, A.R., and Hall, I.M. (2010). BEDTools: a flexible suite of utilities for comparing genomic features. Bioinformatics *26*, 841–842.

Ramirez, F., Bhardwaj, V., Arrigoni, L., Lam, K.C., Gruning, B.A., Villaveces, J., Habermann, B., Akhtar, A., and Manke, T. (2018). High-resolution TADs reveal DNA sequences underlying genome organization in flies. Nat. Commun. *9*, 189.

Rao, S.S., Huntley, M.H., Durand, N.C., Stamenova, E.K., Bochkov, I.D., Robinson, J.T., Sanborn, A.L., Machol, I., Omer, A.D., Lander, E.S., and Aiden, E.L. (2014). A 3D map of the human genome at kilobase resolution reveals principles of chromatin looping. Cell *159*, 1665–1680.

Roberts, A., and Pachter, L. (2013). Streaming fragment assignment for real-time analysis of sequencing experiments. Nat. Methods *10*, 71–73.

Rotelli, M.D., Policastro, R.A., Bolling, A.M., Killion, A.W., Weinberg, A.J., Dixon, M.J., Zentner, G.E., Walczak, C.E., Lilly, M.A., and Calvi, B.R. (2019). A Cyclin A-Myb-MuvB-Aurora B network regulates the choice between mitotic cycles and polyploid endoreplication cycles. PLoS Genet. *15*, e1008253.

Royce, T.E., Carriero, N.J., and Gerstein, M.B. (2007). An efficient pseudomedian filter for tiling microrrays. BMC Bioinformatics *8*, 186.

Rushton, J.J., Davis, L.M., Lei, W., Mo, X., Leutz, A., and Ness, S.A. (2003). Distinct changes in gene expression induced by A-Myb, B-Myb and c-Myb proteins. Oncogene *22*, 308–313.

Schwartz, Y.B., Linder-Basso, D., Kharchenko, P.V., Tolstorukov, M.Y., Kim, M., Li, H.B., Gorchakov, A.A., Minoda, A., Shanower, G., Alekseyenko, A.A., et al. (2012). Nature and function of insulator protein binding sites in the Drosophila genome. Genome Res. *22*, 2188–2198.

Sexton, T., Yaffe, E., Kenigsberg, E., Bantignies, F., Leblanc, B., Hoichman, M., Parrinello, H., Tanay, A., and Cavalli, G. (2012). Three-dimensional folding and functional organization principles of the Drosophila genome. Cell *148*, 458–472.

Sim, C.K., Perry, S., Tharadra, S.K., Lipsick, J.S., and Ray, A. (2012). Epigenetic regulation of olfactory receptor gene expression by the Myb-MuvB/dREAM complex. Genes Dev. *26*, 2483–2498.

Smith, E.M., Lajoie, B.R., Jain, G., and Dekker, J. (2016). Invariant TAD Boundaries Constrain Cell-Type-Specific Looping Interactions between Promoters and Distal Elements around the CFTR Locus. Am. J. Hum. Genet. *98*, 185–201.

Supek, F., Bošnjak, M., Škunca, N., and Šmuc, T. (2011). REVIGO summarizes and visualizes long lists of gene ontology terms. PLoS One *6*, e21800.

Tanaka, Y., Patestos, N.P., Maekawa, T., and Ishii, S. (1999). B-myb is required for inner cell mass formation at an early stage of development. J. Biol. Chem. *274*, 28067–28070.

Trinklein, N.D., Aldred, S.F., Hartman, S.J., Schroeder, D.I., Otillar, R.P., and Myers, R.M. (2004). An abundance of bidirectional promoters in the human genome. Genome Res. *14*, 62–66.

Tsukiyama, T., and Wu, C. (1995). Purification and properties of an ATP-dependent nucleosome remodeling factor. Cell *83*, 1011–1020.

Tsukiyama, T., Becker, P.B., and Wu, C. (1994). ATP-dependent nucleosome disruption at a heat-shock promoter mediated by binding of GAGA transcription factor. Nature *367*, 525–532.

Van Bortle, K., Ramos, E., Takenaka, N., Yang, J., Wahi, J.E., and Corces, V.G. (2012). Drosophila CTCF tandemly aligns with other insulator proteins at the borders of H3K27me3 domains. Genome Res. *22*, 2176–2187.

Van Bortle, K., Nichols, M.H., Li, L., Ong, C.T., Takenaka, N., Qin, Z.S., and Corces, V.G. (2014). Insulator function and topological domain border strength scale with architectural protein occupancy. Genome Biol. *15*, R82.

Vockley, C.M., D'Ippolito, A.M., McDowell, I.C., Majoros, W.H., Safi, A., Song, L., Crawford, G.E., and Reddy, T.E. (2016). Direct GR Binding Sites Potentiate

OPEN
ACCESS
CellPress

Clusters of TF Binding across the Human Genome. Cell *166*, 1269–1281.e1219.

Wen, H., Andrejka, L., Ashton, J., Karess, R., and Lipsick, J.S. (2008). Epigenetic regulation of gene expression by Drosophila Myb and E2F2-RBF via the Myb-MuvB/dREAM complex. Genes Dev. *22*, 601–614.

Werwein, E., Schmedt, T., Hoffmann, H., Usadel, C., Obermann, N., Singer, J.D., and Klempnauer, K.H. (2012). B-Myb promotes S-phase independently of its sequence-specific DNA binding activity and interacts with polymerase delta-interacting protein 1 (Pdip1). Cell Cycle *11*, 4047–4058.

Yang, J., and Corces, V.G. (2011). Chromatin insulators: a role in nuclear organization and gene expression. Adv. Cancer Res. *110*, 43–76.

Yang, L., and Yu, J. (2009). A comparative analysis of divergently-paired genes (DPGs) among Drosophila and vertebrate genomes. BMC Evol. Biol. *9*, 55.

Yang, J., Ramos, E., and Corces, V.G. (2012). The BEAF-32 insulator coordinates genome organization and function during the evolution of Drosophila species. Genome Res. *22*, 2199–2207.

STAR★METHODS

KEY RESOURCES TABLE

REAGENT or RESOURCE	SOURCE	IDENTIFIER
Antibodies		
Anti-histone H3K4me3	Abcam	Cat# ab8580; RRID:AB_306649
Anti-histone H3K27me3	Abcam	Cat# ab6002; RRID:AB_305237
Anti-histone H3K9me3	Abcam	Cat# ab8898; RRID:AB_306848
Rabbit Anti-CP190	Corces laboratory	N/A
Rabbit Anti-BEAF-32	Corces laboratory	N/A
Rabbit Anti-Rpb3 subunit of Pol II	Adelman laboratory	N/A
Rabbit polyclonal Anti-Myb	Botchan laboratory	N/A
Chemicals, Peptides, and Recombinant Proteins		
TRIzol	Invitrogen	Cat#80806
cOmplete, Protease Inhibitor Cocktail	Roche	Cat#19541400
Dynabeads Protein A	Invitrogen	Cat#00324078
RNase A	Invitrogen	Cat#1383185
Proteinase K	Invitrogen	Cat#1250776
Critical Commercial Assays		
RNeasy Mini Kit	QIAGEN	Cat#74104
SuperScript Double-Stranded cDNA Synthesis Kit	Invitrogen	Cat#1225016
PowerUp SYBR Green Master Mix	Applied Biosystems	Ref#100029284
Dual-Color DNA Labeling Kit	NimbleGen	Cat#13678600
SureTag DNA labeling Kit	Agilent	Cat#0006300758
Deposited Data		
Raw and analyzed data	This paper	GSE100143
D. melanogaster reference genome NCBI	Dm3 5.7	ftp://ftp.flybase.net/genomes/Drosophila_melanogaster/dmel_r5.7_FB2008_04/
Insulator proteins ChIP-chip	modENCODE Consortium et al., 2010	http://www.modencode.org/
Experimental Models: Organisms/Strains		
D. melanogaster: *Df(1)MH107, w^{1118}/FM7i, P{w[+mC] = ActGFP}JMR3*	Bloomington Drosophila Stock Center	Cat#30559; RRID:BDSC_30559
D. melanogaster: *$y^{1}w^{67c23}$*	Bloomington Drosophila Stock Center	Cat#6599; RRID:BDSC_6599
D. melanogaster: *w[*]; $E2f2^{76Q1}$, cn^{1}, bw^{1}/ CyO, P(ry[+t7.2] = ftz/lacB)E3*	Bloomington Drosophila Stock Center	Cat#7436; RRID:BDSC_7436
D. melanogaster: *w[*]; Df(2L)G5.1, dpy[ov^{1}] b^{1}/CyO, P(ry[+t7.2] = ftz/lacB)E3*	Bloomington Drosophila Stock Center	Cat#7437; RRID:BDSC_7437
Oligonucleotides		
Primers for quantitative real-time PCR, see Table S5	This paper	N/A
Software and Algorithms		
ArrayStar (Version 12.0.0)	DNASTAR, Inc.	https://www.dnastar.com/
Agilent Feature Extraction (Version 9.5.1)	Agilent	https://www.agilent.com
Agilent Genomics Workbench (Version 7.0)	Agilent	https://www.agilent.com
JASPAR	Mathelier et al., 2016	http://jaspar.genereg.net/
MEME suite	Bailey et al., 2009	http://meme-suite.org/tools/meme
WebLogo	Crooks, et al., 2004	http://weblogo.berkeley.edu/logo.cgi
PRISM Version 8	Graphpad	https://www.graphpad.com/scientific-software/prism/
Image Processing and Analysis in Java	ImageJ	https://imagej.nih.gov/ij/index.html

LEAD CONTACT AND MATERIALS AVAILABILITY

Further information and requests for resources and reagents should be directed to and will be fulfilled by the Lead Contact, J. Robert Manak (john-manak@uiowa.edu). This study did not generate new unique reagents.

EXPERIMENTAL MODEL AND SUBJECT DETAILS

Drosophila strains

The Myb^{MH107} line (*Df(1)MH107*, w^{1118}*/FM7i, P{w[+mC] = ActGFP}JMR3*) is previously described (Manak et al., 2002) and is a null allele generated by mobilizing a nearby P-element which removes the 5′ end of Myb as well as a non-essential gene (*alkB*), and all observed *Myb* mutant-related phenotypes (including lethality) can be rescued through ubiquitous expression of a Myb cDNA in the mutant (Manak et al., 2007). Myb^{MH107} was outcrossed into the y^1w^{67c23} laboratory line used extensively in our laboratory as a control stock for a minimum of 7 generations to minimize genetic background issues. The *Myb* mutation is located on the X chromosome and thus carried in heterozygous females also carrying a GFP-marked balancer (with males of the line only carrying the GFP-marked balancer); this required selection of GFP- animals (males) for our mutant analysis, and thus only males were used for controls. The $E2f2^{76Q1}$ mutant line (*w[*]; E2f2*^{76Q1}*, cn*^{1}*, bw*^{1}*/CyO, P(ry[+t7.2] = ftz/lacB)E3*) and the deficiency stock that takes out *E2f2* (*w[*]; Df(2L)G5.1, dpy[ov*^{1}*] b*^{1}*/CyO, P(ry[+t7.2] = ftz/lacB)E3*) were obtained from the Bloomington *Drosophila* Stock Center and used in combination ($E2f2^{76Q1}$ over deficiency) for *E2f2* mutant analyses. Given that *E2f2* mutations are on the second chromosome, both males and females were used for the mutant and control analysis. Stocks were maintained at 25°C in malt-based media (Archon Scientific and University of Iowa Biology Department Fly Kitchen).

METHOD DETAILS

Microarray gene expression analysis

Drosophila wing imaginal discs were dissected from *Myb* and yw^{67} third instar in 1X PBS (pH 7.4) and transferred to TRIzol reagent (Invitrogen). Total RNA was isolated and further purified utilizing the RNeasy Mini Kit (QIAGEN). Double-stranded cDNA was generated using the SuperScript Double-Stranded cDNA Synthesis Kit with random hexamer (Invitrogen). cDNA was labeled using Cy3-coupled random nonamers (Dual Color Labeling Kit, NimbleGen) and three biological replicates with 3 to 4 technical replicates each were hybridized in the Gene Expression 12 × 135K Array (NimbleGen). After hybridization for 20 hours, the arrays were scanned on an Axon GenePix 4200A microarray scanner (Molecular Devices). Raw data (Pair files) were normalized in ArrayStar software version 12.0.0 (DNASTAR, Inc, Madison, WI). The robust multichip analysis (RMA) algorithm was used for background correction, quantile normalization, and median polish summarization. A Student's t test corrected for multiple testing with the Benjamini and Hochberg false discovery rate (FDR) method was calculated for each experiment. Transcripts were considered differentially expressed if showing an FDR-adjusted *P value* of less than 0.001 and a minimum absolute signal intensity of 500 for at least one sample (Table S2). See Table S2 for r^2 replicate correlations.

Tiling array: 3rd instar larvae were dissected in 1X PBS (pH 7.4). 10 μg of total RNA was extracted from yw^{67} and *Myb* mutant 3rd instar larval wing discs using TRIzol (Invitrogen) and purified with RNeasy Mini Kit (QIAGEN). cDNA was generated using the Double-Stranded cDNA Synthesis Kit with random hexamer (Invitrogen). RNA and cDNA quality was analyzed using Experion RNA and DNA analysis kits (Bio-Rad), respectively. 1 μg of cDNA from each genotype was labeled using either Cy3 and Cy5 random nanomers. In order to optimize comparisons between control and mutant, a competitive hybridization was performed with equal amounts of differentially labeled cDNA (15 μg) for two biological replicates of both yw^{67} and *Myb*-. Hybridization and scanning of the arrays were performed following the manufacturer's standard protocol (https://www.roche.com/) The array set [designed by JR Manak; see Nien et al. (2011)] utilizes two 2.1 million feature 50-mer oligonucleotide probe microarrays using Genome Release 5 with a median probe spacing of 33 bp. Repeat rich sequences such as heterochromatin and transposons were included in the design. For this reason, up to close 100 matches per sequence were tolerated. Probe data, probe files (.pair files) were run in NimbleScan software to produce processed scaled log2 data (.gff files) used for visualization.

RNA-seq analysis

Total RNA was isolated from yw^{67} control (3 biological replicates) and *Myb* mutant (5 biological replicates) 3rd instar mesothoracic wing discs. cDNA was generated from polyadenylated mRNA captured with oligo-dT beads. The cDNA was then sequenced on two lanes (all 8 samples) of an Illumina HiSeq 4000 Genome Sequencer (Iowa Institute of Human Genetics). The resulting read data was trimmed for adaptor sequences using Trimmomatic-0.32 (Bolger et al., 2014) and the trimmed reads were mapped to gene exons of the *D. melanogaster* 2008 build, dmel r5.7 version (ftp://ftp.flybase.net/genomes/Drosophila_melanogaster/dmel_r5.7_FB2008_04/fasta/dmel-2L-exon-r5.7.fasta.gz), using bowtie version 2.1.0 (Langmead and Salzberg, 2012). Reads from the two lanes were then combined to generate one alignment file for each of the 8 samples using bowtie. On average, ~81% of reads aligned to the genome. The eXpress tool was used to generate uniquely mapped read counts for each exon of a gene (Roberts and Pachter, 2013). The sum of unique read counts for each gene was generated using a custom python script. Due to the high correlation among biological replicates, all replicates for each the control and *Myb* mutant were used in the gene expression analysis. The

DESeq2 package in R was used to compute normalized fold-change and *P value* for each gene, comparing *Myb* mutant to control (Love et al., 2014). This identified a total of 3,410 significantly differentially expressed genes with an adjusted *P value* of < 0.05. See Supplemental Data for mapping statistics.

Gene Ontology analysis

Gene Ontology analysis was performed using the PANTHER analysis tool (Mi et al., 2019). REVIGO (Supek et al., 2011) was used to summarize the GO terms.

Chromatin immunoprecipitation

Wing imaginal discs were dissected from *yw*^{67} and *Myb* male 3rd instar larvae in cold 1X PBS with cOmplete, Protease Inhibitor Cocktail (Roche). The tissue was fixed with 1.8% formaldehyde and incubated for 15 mins at room temperature on a rotating wheel. Crosslinking was stopped by adding glycine to a final concentration of 125mM in 0.1% PBS-Triton and incubated for 5 mins at room temperature. Samples were washed twice with lysis buffer (50 mM HEPES, pH 7.8, 10 mM EDTA, 0.5% *N*-lauroylsarcosine, and Roche cOmplete Protease Inhibitor Cocktail), snap-frozen in liquid nitrogen and stored at -80°C. Chromatin for each immunoprecipitation experiment was prepared from a total of 800 wing imaginal discs and sonicated (30 pulses of 15 s ON/ 15 s OFF, high energy setting) in a Bioruptor (Diagenode) resulting in an average DNA fragment size of 400 bp. Samples were pre-cleared by adding 20 μl of Dynabeads® Protein A (Invitrogen) equilibrated in IP buffer (16.7 mM Tris-HCl, pH 8.0, 167 mM NaCl, 10 mM EDTA, 1.1% Triton, 0.1% SDS + 0.5% BSA) for 2 hr at 4°C. Immunoprecipitation was performed at 4°C overnight utilizing antibodies for H3K4me3 (ab8580; Abcam), H3K27me3 (ab6002; Abcam), H3K9me3 (ab8898; Abcam), anti-CP190 and anti-BEAF-32 were cordially provided by Dr. Victor Corces (Emory University), anti-Myb and anti-Rpb3 subunit of Pol II were kindly provided by Dr. Michael Botchan (University of California, Berkley) and Dr. Karen Adelman (NIH), respectively. The immunocomplexes were recovered by adding 20 μl of Dynabeads® Protein A (Invitrogen) (previously equilibrated) for 3 hr at 4°C and washed with the following buffers: 1X for 10 mins with 1 mL of Low Salt Buffer (140 mM NaCl, 10 mM Tris-HCl, pH 8.0, 1 mM EDTA, 0.1% SDS, 1% Triton X-100, 0.1% Sodium-deoxycholate); 5X for 10 mins with 1 mL of High Salt Buffer (500 mM NaCl, 10 mM Tris-HCl, pH 8.0, 1 mM EDTA, 0.1% SDS, 1% Triton X-100, 0.1% Sodium-deoxycholate) and once with 1 mL of LiCl buffer (250 mM LiCl, 10 mM Tris-HCl, pH 8.0, 1mM EDTA, 0.5% NP-40, 0.5% Sodium-deoxycholate); and 2X for 10 mins with TE Buffer. DNA was released from the beads by adding elution buffer (50 mM Tris-HCl, pH 8.0, 10 mM EDTA, 1% SDS) and incubating the samples at 65°C for 15 mins with occasional vortexing. TE Buffer was added to the eluted sample and input following by incubation in the presence of RNase A (Invitrogen) was to a final concentration of 0.1 mg/mL and incubated at 37°C for 1 hr. Proteinase K (Invitrogen) was added to a final concentration of 0.5 mg/mL and incubated at 37°C overnight. The samples were then moved to 65°C and incubated for 6hrs to reverse the crosslinks. Finally, DNA was phenol/chloroform extracted and ethanol precipitated.

ChIP-chip analysis

DNA from the ChIP experiments were differentially labeled with Cy3- and Cy5-coupled random nonamers (SureTag DNA Labeling Kit, Agilent), hybridized onto a custom *Drosophila* tiled genomic microarray utilizing the dm3 assembly (G4123A, SurePrint G3 Custom CGH Microarray 1x1M - Agilent) containing 60-mer probes spanning the whole genome including repeat sequences. Raw data was extracted utilizing Agilent feature extraction V9.5.1 (Agilent). Data was normalized by blank subtraction, inter- and intra-array (dye-bias) median normalization using Agilent Genomics Workbench V7.0 (Agilent). For Myb ChIP, bound regions were detected using the Whitehead Per-Array Neighborhood Model in which two consecutive probes had to be at a maximal distance of 1000bp with an average *P value* of less than 0.05 between the central probe and at least one of its neighbors. Peaks were called if four or more significant probes were at a maximum distance of 300bp.

H3K27me3 signal was calculated by extracting probe data expanding published *Drosophila* H3K27me3 domains (Sexton et al., 2012). We collected signal intensities 10,000bp upstream and downstream of domain boundaries. We also obtained signal intensities 5,000bp upstream and downstream of the center of domains. Averages were calculated utilizing a sliding window approach with a window length of 10bp moving at a 1bp rate. To calculate whether there was a statistically significant difference in average probe intensities between controls and *Myb* mutants within the H3K27me3 domains, we utilized all probe signals contained within the domains for both control and *Myb* mutant, and performed a Student's t test.

Motif discovery

Sequences underlying the start and end of Myb peaks present at promoter regions of genes (TSS ± 500bp) were collected. Myb motif Position Weight Matrices (PWM) were obtained from the JASPAR database (Mathelier et al., 2016) and a Markov model background of 7bp was estimated utilizing the fasta-get-markov feature from the MEME suite (Bailey et al., 2009). Overrepresented sequences showing a $p < 0.01$ were retained. The logo diagram was created with these sequences utilizing WebLogo (Crooks et al., 2004) with default settings. We utilized FIMO (Grant et al., 2011) to detect individual Myb and E2f2 motifs at promoters, defined as ± 500 bp from TSS, of upregulated genes. Previously published Myb motif (YAACKG) and E2f2 (TTSSSSS) motif were used to scan input sequences at $p < 0.001$.

RNA polymerase analysis

Gene categories 1 and 2 (Figure 1) were identified based on whether the median RNA Pol II signal (all probes within the −30 to 300 bp promoter region) was either higher or lower in the *Myb* mutant relative to control with a minimum gene length of 1,000 bp. Probe signal intensities from the RNA polymerase II ChIP-chip control and *Myb* mutant experiments were extracted for direct Myb target genes called as significantly downregulated by both microarray and RNA-seq. We specifically used probe data from −2000 bp relative to TSS to +5000 bp relative to TSS. We used a sliding window approach to calculate the median of signal intensities for all genes in a category using a window length of 100 bp moving 1bp at a time. We then took the median signal intensity for all genes at each base position. Negative strand genes were flipped and matched with positive strand genes. To determine whether the RNA Pol II signal at the promoter and across the gene body was statistically significant between the controls and *Myb* mutants, we performed a Mann-Whitney U test using all probes within the respective regions. RNA Pol II profile graphs were smoothed by applying a 900 bp average sliding window.

Upregulated genes for H3K4 and H3K27 significance analysis

Normalized signal intensities for control and *Myb* mutant ChIP-chip H3K27me3 array data associated with a 2000 bp interval (+200 bp from TSS to +2200 bp) were collected. Similarly, the normalized signal intensities from the H3K4me3 array data associated with a 2000 bp interval (from TSS to +2000 bp) were also collected. A Student's t test was used to assess whether the difference in mean signal intensity between control and mutant was significant for each gene and a *P value* was generated. Multiple comparison *P value*s were adjusted for false discovery (FDR) and $p < 0.05$ was considered significant.

Wig files for visualization of ChIP-chip and tiling array data in UCSC were prepared after smoothing probe signal intensity utilizing a pseudomedian algorithm previously described (Royce et al., 2007). Parameters chosen were the following: span = 3 and Mohanan-modified algorithm. Wig files for the RNA-seq data were generated by first converting the bam alignment files into bed format using bedtools (Quinlan and Hall, 2010). Next, the bed files were converted into wig format based on the wig specifications from UCSC using a python script.

Overlaps of Myb peaks with genomic elements

Overlap of Myb peaks with genomic features was assessed based on the following ranking: promoters (defined as −200bp from the TSS), exons, introns and intergenic regions. Genome sequence information was obtained from the *Drosophila* UCSC dm3 assembly. Publicly available peak insulator data was obtained from the modENCODE project (Contrino et al., 2012; modENCODE Consortium et al., 2010; Nègre et al., 2011). Peaks were considered overlapping if a Myb peak was within ± 500bp of an insulator peak. The insulator and anchor point datasets used were the following (modENCODE Consortium et al., 2010): CTCF_N_S2 (DCCid: modENCODE_913), Su(Hw) (DCCid: modENCODE_27, mod(mdg4) (DCCid: modENCODE_24, GAF (DCCid: modENCODE_23), BEAF-32 (DCCid: modENCODE_21), CP190 (DCCid: modENCODE_22), Pc (DCCid: modENCODE_3791). Rad21 binding site data was obtained from Van Bortle et al. (2014). For the overlap of Myb peaks with DPGs, we considered head to head genes as a DPG if the negative strand gene TSS was within or equal to 1,000 bp from the TSS of the positive strand gene (Trinklein et al., 2004; Yang and Yu, 2009). If a gene was present in multiple pairs, each was considered a DPG provided the distance between TSSs was < or = 1,000bp. Histone clusters were not considered in this analysis. Myb was considered to overlap a DPG if a peak was present within 500 bp downstream of either TSS or between the genes. For the overlap of Myb peaks with TAD boundaries (Sexton et al., 2012), a peak was considered positive if it was present within 5 kb of the TAD boundary.

Random permutation testing

Significance testing of Myb overlaps was determined by first assessing the peaks uniquely mapping to various genomic elements (e.g., anchors, insulator protein peaks, DPGs, etc.). We then estimated the random distribution of expected Myb peak binding by permuting peaks and elements 1000 times using bedtools v2.26.0 (Quinlan and Hall, 2010). For each analysis, we maintained the same number of peaks, the peak size, and their respective chromosomes. Overlapping Myb peaks and elements were assessed, and a z-test was used to determine significance between the observed data and the permuted distribution (Vockley et al., 2016).

Quantitative real-time PCR analyses

For ChIP-quantitative real-time PCR, the mean Ct value of three technical replicates for each of the two biological replicates was calculated, followed by the mean of the biological replicates. Enrichment of each peak was determined as the fold enrichment of the region of interest over a reference region devoid of an insulator peak or H3K4me3/ H3K27me3 peak. For qRT-PCR, RNA from wing discs was isolated using the RNeasy Plus mini kit (QIAGEN) according to manufacturer's instructions. cDNA was prepared from 0.5-1 μg total RNA using High-Capacity RNA-to-cDNA kit (Applied Biosystems) and the mean Ct value of three technical replicates for each of the three biological replicates was calculated after normalizing to housekeeping gene *rp49*, followed by the mean of the biological replicates. Fold change values were calculated relative to control. Samples were amplified using the PowerUp SYBR Green Master Mix (Applied Biosystems) and quantified using an Applied Biosystems 7900HT real-time PCR machine according to the manufacturer's protocols. Statistical significance was calculated using a two-tailed unpaired Student's t test.

Western blot analysis

Wing discs from 3rd instar wandering larvae were dissected in PBS and lysed in SDS sample buffer. The homogenized discs were heated at 95°C for 5 minutes and immediately fractionated with SDS-PAGE, transferred onto a 0.45um nitrocellulose membrane (GE Healthcare Life Sciences) and probed overnight at 4°C with antibodies against CP190 (1:1000; Corces laboratory), BEAF-32 (Blanton et al., 2003) (1:100; DSHB, University of Iowa) and anti-alpha Tubulin (1:1000; AA4.3-c, DSHB, University of Iowa). Blots were developed with the Pierce ECL substrate (ThermoFisher Scientific) following the manufacturer's recommendations.

QUANTIFICATION AND STATISTICAL ANALYSIS

Data were analyzed with a two-tailed unpaired, Student's t test, Mann-Whitney U test, Pearson correlation analysis, and random permutation testing as described above. Error bars for quantitative real-time PCR analysis represent the mean ± SEM. Two-tailed *P value*s of < 0.05 were considered the cutoff for statistical significance unless otherwise indicated.

DATA AND CODE AVAILABILITY

Gene expression data (both microarray and RNA-seq) and ChIP-chip data are available at the GEO database, accession number GSE100143.

Cell Reports, Volume *30*

Supplemental Information

The Dm-Myb Oncoprotein Contributes to Insulator Function and Stabilizes Repressive H3K27me3 PcG Domains

Juan F. Santana, Mrutyunjaya Parida, Abby Long, Joshua Wankum, Anthony J. Lilienthal, Krishna M. Nukala, and J. Robert Manak

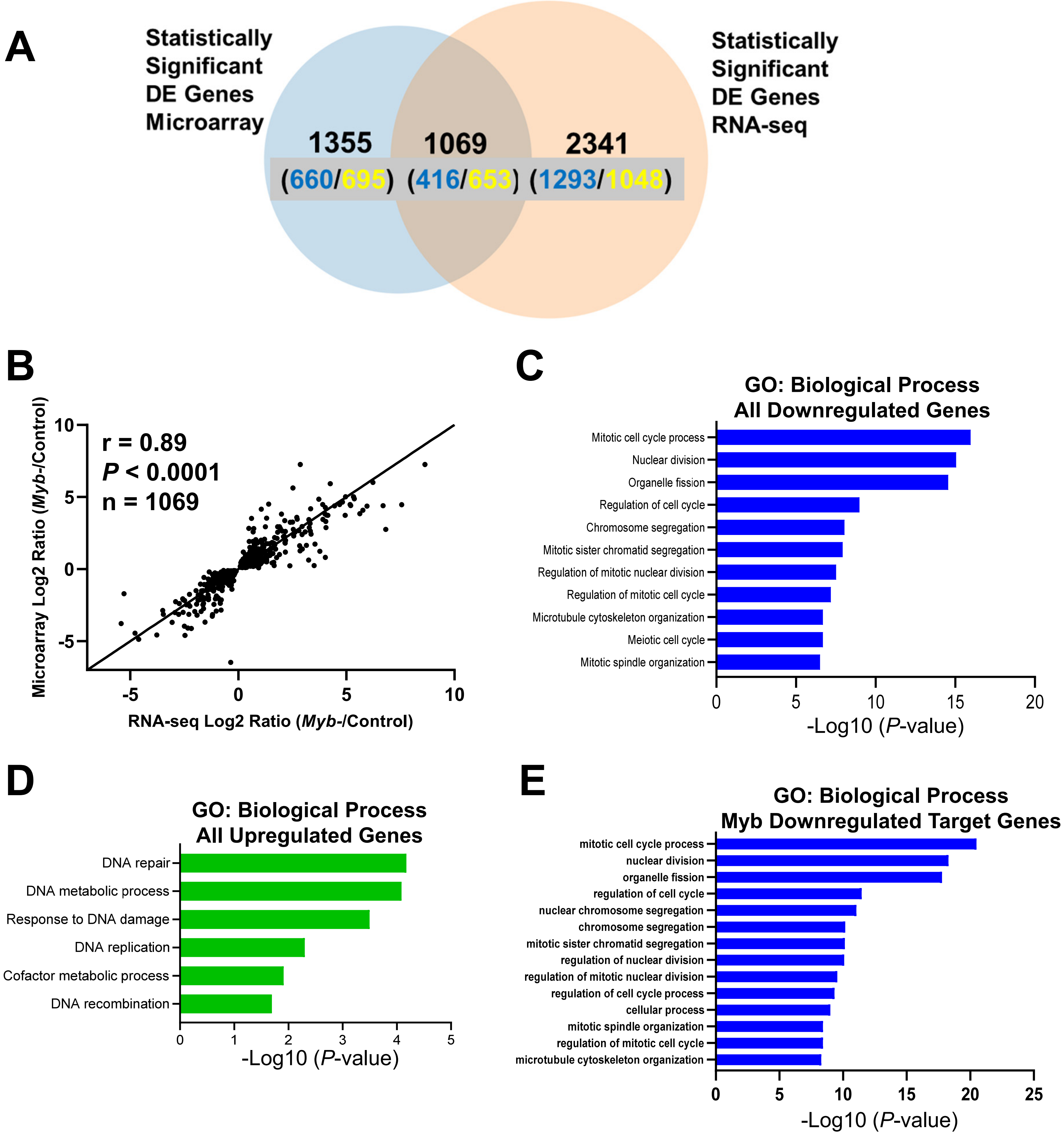

Figure S1. Comparison of gene expression microarray with RNA-seq data and gene ontology analysis of differentially expressed genes in the *Myb* mutant. Related to Figure 1. (A) Venn diagram comparison of statistically significant differentially expressed genes (control vs. *Myb-*) called by array ($P < 0.001$) and RNA-seq ($P < 0.05$). Numbers in blue denote downregulated genes in *Myb* mutant relative to control; numbers in yellow denote upregulated genes in *Myb* mutant relative to control. DE = differentially expressed. (B) Pearson correlation of the overlap of statistically significant differentially expressed genes called by both array and RNA-seq. (C) List of enriched biological processes associated with downregulated genes in the *Myb* mutant relative to control. (D) List of enriched biological processes associated with upregulated genes in the *Myb* mutant relative to control. (E) List of enriched biological processes associated with downregulated genes that are direct Myb targets in *Myb* mutant relative to control.

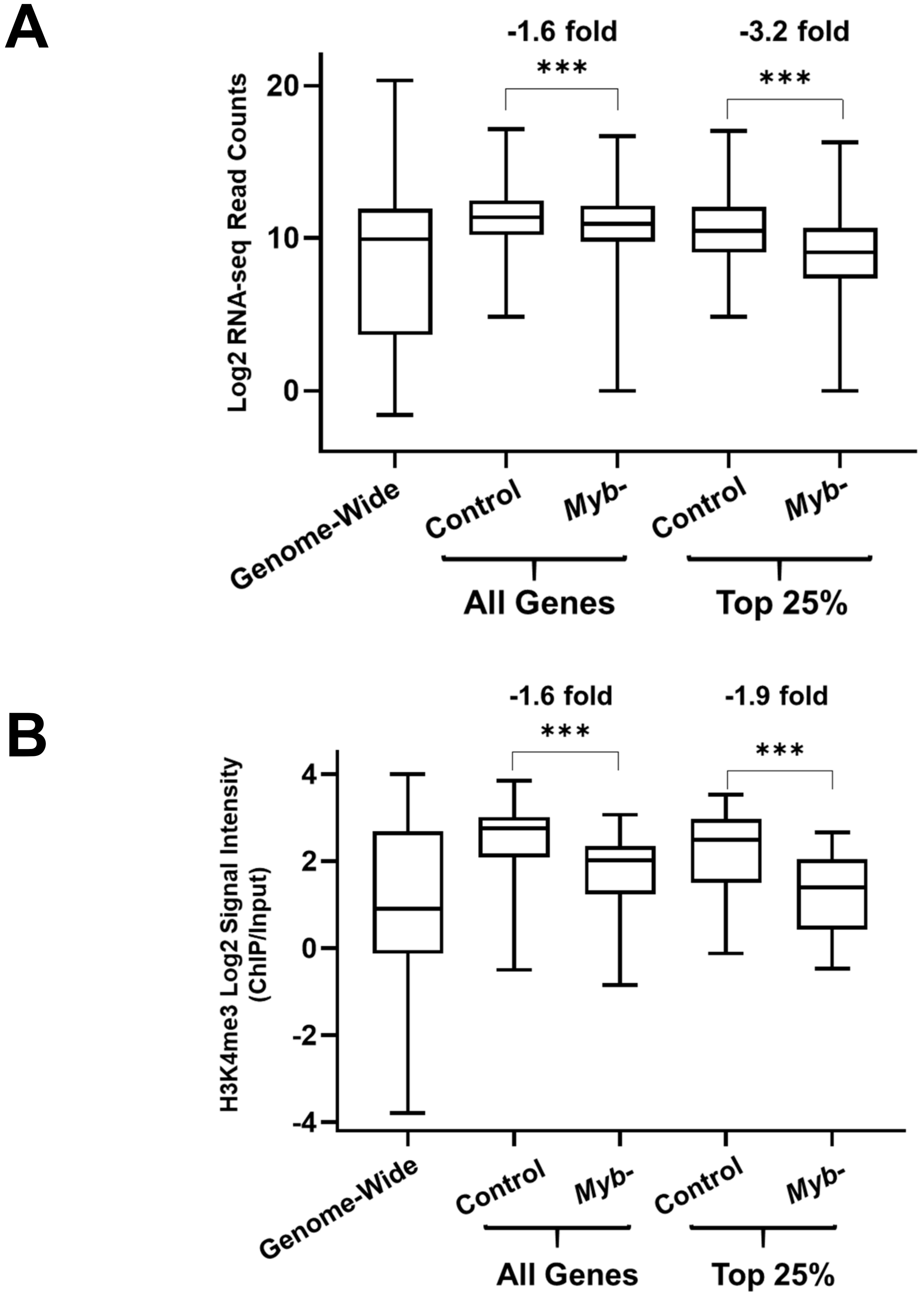

Figure S2. Direct Myb targets are highly expressed and potentiated by Myb. Related to Figure 1. (A) Comparison of expression levels of direct Myb-potentiated target genes in control and *Myb* mutants compared to gene expression levels genome-wide using RNA-seq data. (B) Comparison of H3K4me3 signal from TSS to 500 bp downstream for all direct Myb target genes in control and *Myb* mutants compared to genes genome-wide. Note that genes potentiated by Myb in (A) have higher median expression levels compared to genome-wide gene expression and that the absence of *Myb* leads to a significant reduction of gene expression (-1.6 median fold change). Also shown is the upper quartile (top 25%; highest fold change difference between *Myb* mutant and control) with a -3.2 median fold change. Reduced expression levels also correlate with reduced H3K4me3 (B; -1.6 median fold change between control and *Myb* mutant; -1.9 median fold change between control and *Myb* mutant for upper quartile genes). Boxes represent interquartile range (25th to 75th percentiles); whiskers represent min to max values; lines within boxes represent medians. All downregulated genes called as statistically significant by both array and RNA-seq. Genome-wide expression levels calculated using all annotated genes. List of genes used for (A) same as genes used for (B). ***$P < 0.0001$ for all comparisons, Mann-Whitney U test.

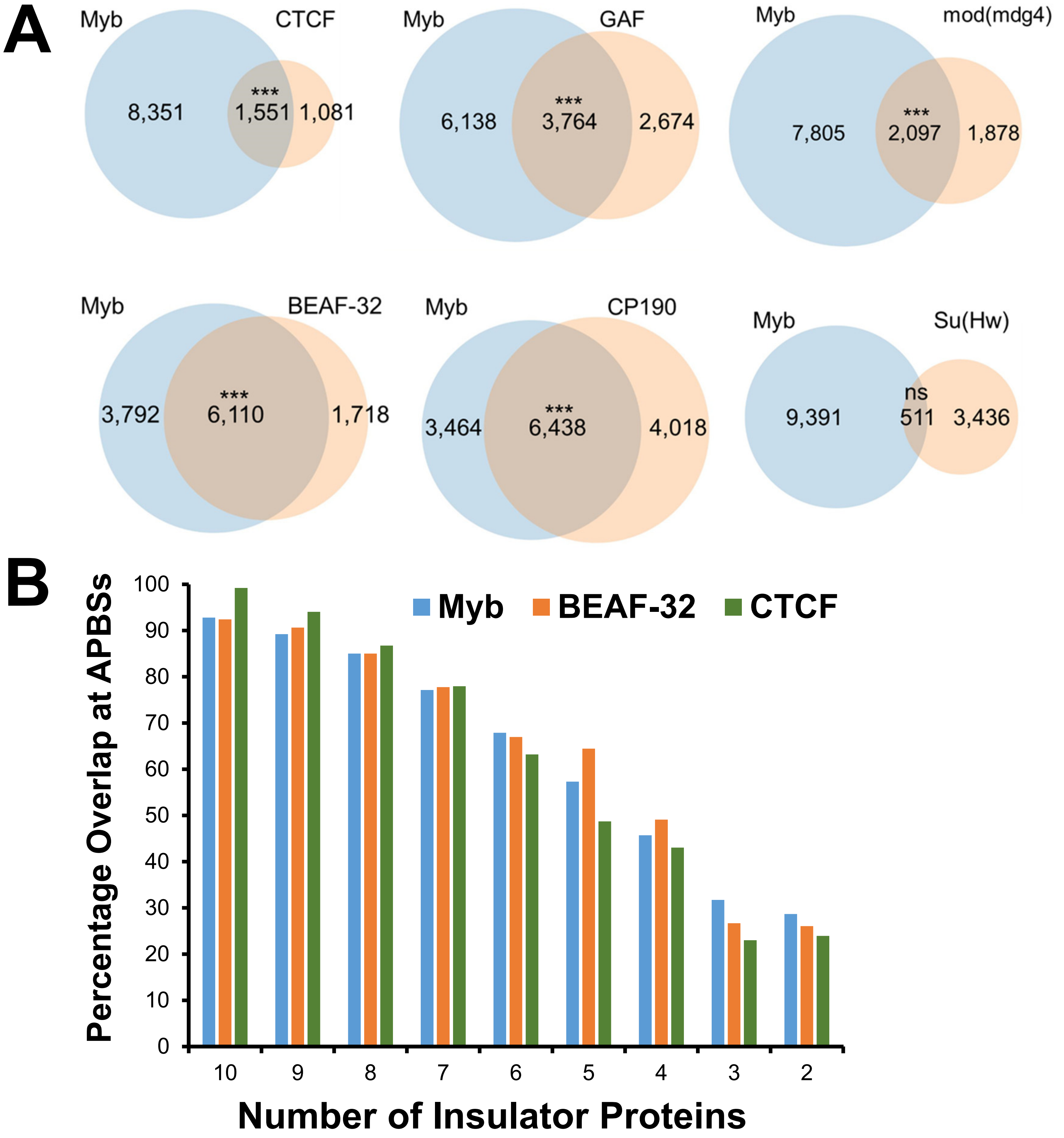

Figure S3. Overlap of Myb binding peaks with insulator protein peaks and high-occurrence architectural protein binding sites (APBS) genome-wide. **Related to Figure 2.** (A) Myb peaks show a statistically significant overlap with BEAF-32 (3.3-fold enrichment, $P < 2.15 \times 10^{-309}$), CP190 (2.8-fold enrichment, $P < 2.15 \times 10^{-309}$), dCTCF (2.3-fold enrichment, $P = 1.73 \times 10^{-132}$), GAF (2.6-fold enrichment, $P < 2.15 \times 10^{-309}$), and mod(mdg4) (2.4-fold enrichment, $P = 1.61 \times 10^{-174}$) peaks but show a negative enrichment for Su(Hw) peaks (0.48-fold enrichment, $P = 1.67 \times 10^{-55}$) and thus non-significant overlap. (B) Co-localization of Myb, BEAF-32, or dCTCF with increasing numbers of insulator proteins dCTCF, BEAF-32, Su(Hw), CP190, mod(mdg4), DREF, Chromator, l(3)mbt, TF3C, CAPH2. Note that colocalization is higher at sites when a maximal number of insulator proteins is present (10) and decreases at sites where fewer insulator proteins cluster. Binding site data for insulators obtained from modENCODE (see Results for sources). All comparisons, random permutation test. *** = statistical significance. ns = non-significance.

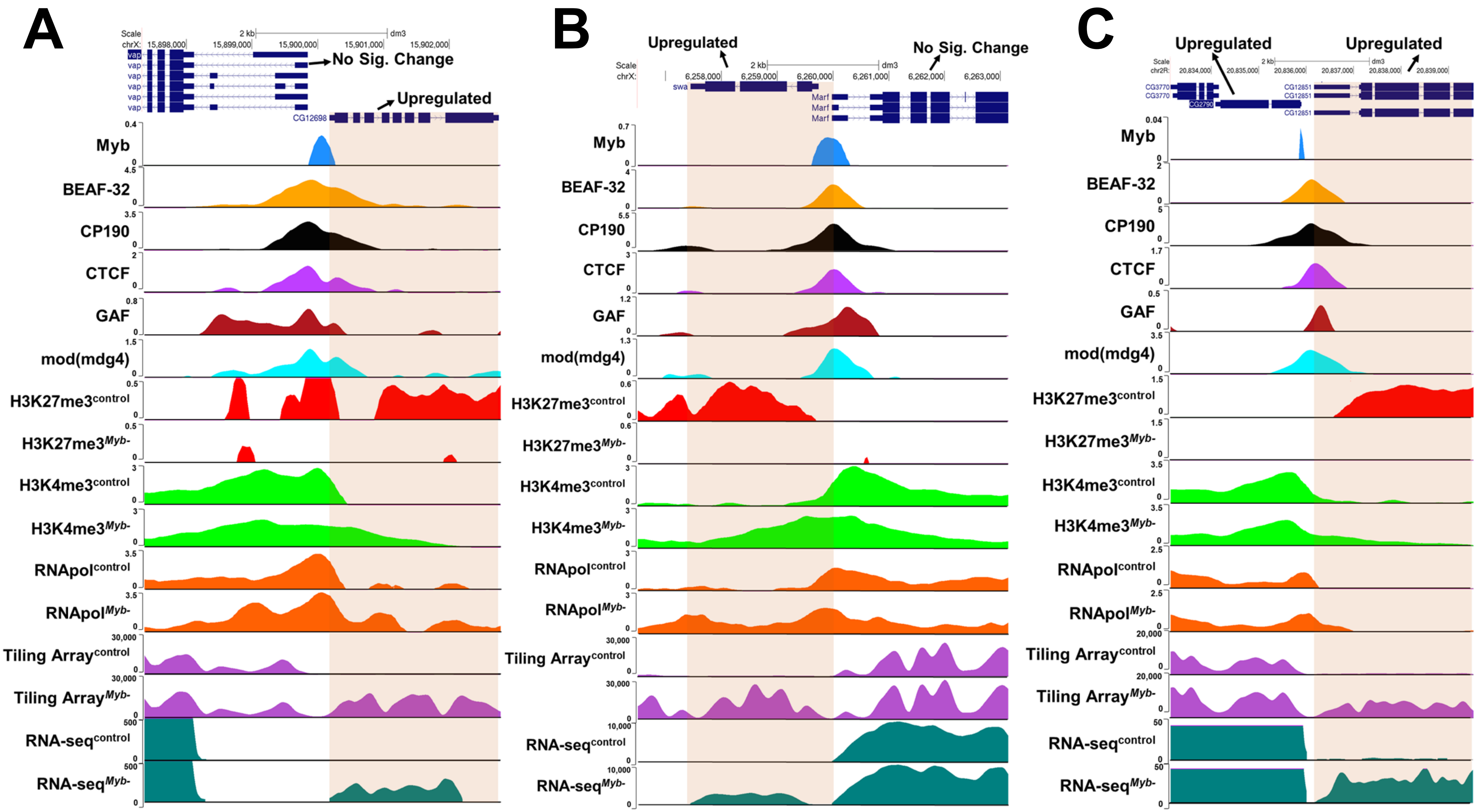

Figure S4. Additional examples of DPG-associated genes upregulated in Myb mutants. Related to Figure 2. (A) Loss of Myb binding between genes results in reduction of H3K27me3 and spreading of H3K4me3, with upregulation of *CG12698* (153 fold array, $P = 8.66 \times 10^{-32}$; 7.2-fold RNA-seq, $P = 1.08 \times 10^{-35}$). (B) Loss of Myb binding between genes results in reduction of H3K27me3 and spreading of H3K4me3, with upregulation of *swa* (64-fold array, $P = 1.26 \times 10^{-27}$; 74-fold RNA-seq, $P < 0.0001$). (C) Loss of Myb binding between genes results in reduction of H3K27me3 and spreading of H3K4me3, with upregulation of both *CG2790* (1.1-fold array, $P = 4 \times 10^{-5}$; 1.1-fold RNA-seq, $P < 0.05$) and *CG12851* (13-fold array, $P = 4.9 \times 10^{-19}$; 18-fold RNA-seq, $P = 1.6 \times 10^{-147}$).

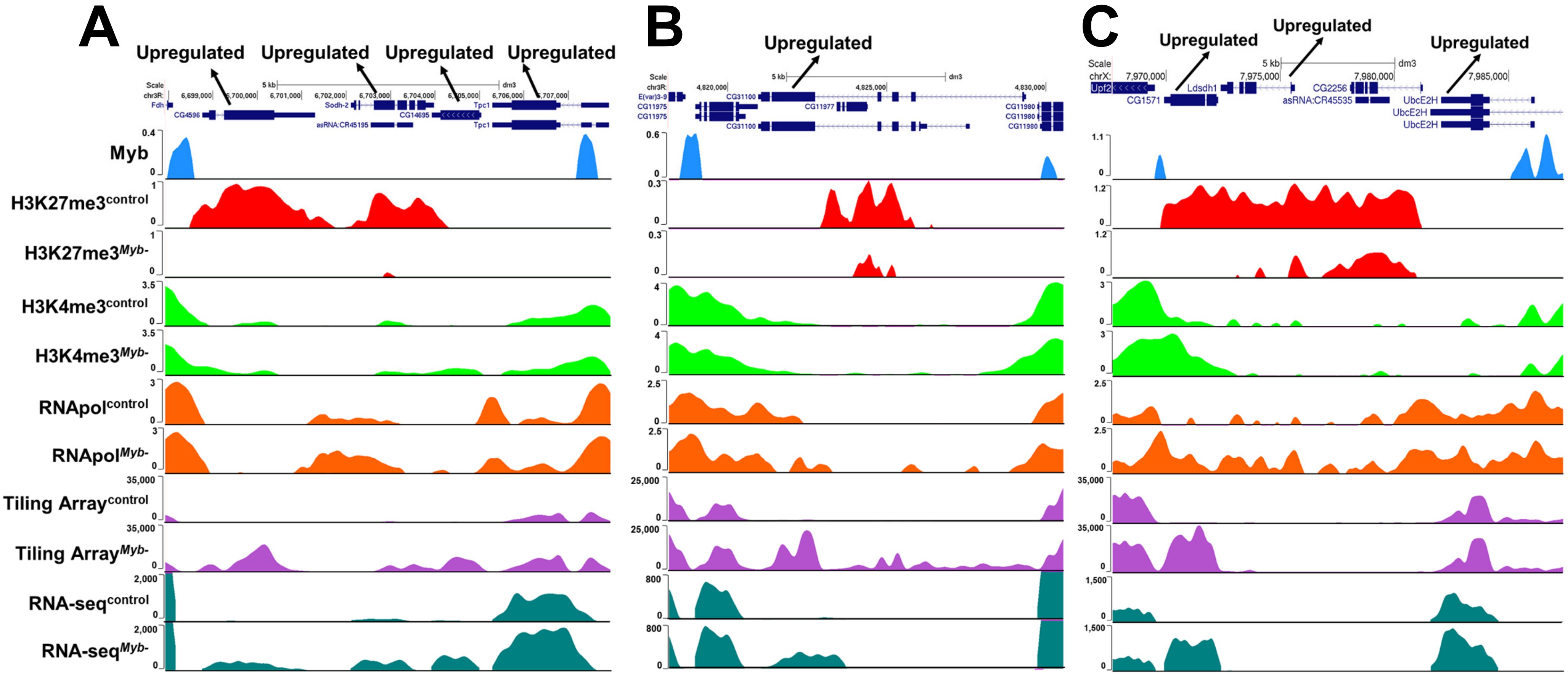

Figure S5. Additional examples of H3K27me3 domains demarcated by Myb. Related to Figure 3. (A) Loss of Myb binding results in reduction of H3K27me3, with upregulation of *CG4596* (22-fold array, $P = 5.6 \times 10^{-20}$; 36-fold RNA-seq, $P = 8.36 \times 10^{-166}$), *Sodh-2* (4.9-fold array, $P = 8.69 \times 10^{-14}$; 4.3-fold RNA-seq, $P = 1.93 \times 10^{-41}$), *CG14695* (14-fold array, $P = 1.69 \times 10^{-22}$; 16-fold RNA-seq, $P = 5.27 \times 10^{-135}$) and *Tpc1* (1.4-fold array, $P = 1.85 \times 10^{-11}$; 1.5-fold RNA-seq, $P = 7.78 \times 10^{-22}$) (B) Loss of Myb binding results in reduction of H3K27me3 and spreading of H3K4me3, with upregulation of *CG31100* (14-fold array, $P = 5.68 \times 10^{-26}$; 4-fold RNA-seq, $P = 4.09 \times 10^{-16}$). (C). Loss of Myb binding results in reduction of H3K27me3 and spreading of H3K4me3, with upregulation of *CG1571* (153-fold array, $P = 5.77 \times 10^{-26}$; 397-fold RNA-seq, $P < 0.0001$), *CG2254* (2.4-fold array, $P = 8.01 \times 10^{-11}$; 3-fold RNA-seq, $P = 6 \times 10^{-10}$), and *UbcE2H* (1.1-fold array, $P = 2.4 \times 10^{-3}$; 1.4-fold RNA-seq, $P = 8.33 \times 10^{-7}$).
